# Diagnostic accuracy of ultrasound in the diagnosis of Placenta accreta spectrum**:** systematic review and meta-analysis

**DOI:** 10.1186/s12884-023-05675-6

**Published:** 2023-05-15

**Authors:** Ahmed M. Maged, Akmal El-Mazny, Nada Kamal, Safaa I. Mahmoud, Mona Fouad, Noura El-Nassery, Amal Kotb, Wael S. Ragab, Asmaa I. Ogila, Ahmed A. Metwally, Yossra Lasheen, Radwa M. Fahmy, Maha Katta, Eman K. Shaeer, Noha Salah

**Affiliations:** 1 Department of Obstetrics and Gynecology, Kasr Al-Ainy Hospital, Cairo University, Giza, Egypt; 2 Department of Obstetrics and Gynecology, Beni-Suef University, Beni-Suef, Egypt; 3 Department of Obstetrics and Gynecology, Fayoum University, Fayoum, Egypt

**Keywords:** Morbidly adherent placenta, Placenta accreta, Placenta accreta spectrum, Placenta increta, Placenta percreta, Prenatal ultrasound diagnosis

## Abstract

**Objective:**

To evaluate the diagnostic accuracy of ultrasound and in the diagnosis of Placenta accreta spectrum (PAS).

**Data sources:**

Screening of MEDLINE, CENTRAL, other bases from inception to February 2022 using the keywords related to placenta accreta, increta, percreta, morbidly adherent placenta, and preoperative ultrasound diagnosis.

**Study eligibility criteria:**

All available studies- whether were prospective or retrospective- including cohort, case control and cross sectional that involved prenatal diagnosis of PAS using 2D or 3D ultrasound with subsequent pathological confirmation postnatal were included. Fifty-four studies included 5307 women fulfilled the inclusion criteria, PAS was confirmed in 2025 of them.

**Study appraisal and synthesis methods:**

Extracted data included settings of the study, study type, sample size, participants characteristics and their inclusion and exclusion criteria, Type and site of placenta previa, Type and timing of imaging technique (2D, and 3D), severity of PAS, sensitivity and specificity of individual ultrasound criteria and overall sensitivity and specificity.

**Results:**

The overall sensitivity was 0.8703, specificity was 0.8634 with -0.2348 negative correlation between them. The estimate of Odd ratio, negative likelihood ratio and positive likelihood ratio were 34.225, 0.155 and 4.990 respectively.

The overall estimates of loss of retroplacental clear zone sensitivity and specificity were 0.820 and 0.898 respectively with 0.129 negative correlation. The overall estimates of myometrial thinning, loss of retroplacental clear zone, the presence of bridging vessels, placental lacunae, bladder wall interruption, exophytic mass, and uterovesical hypervascularity sensitivities were 0.763, 0.780, 0.659, 0.785, 0.455, 0.218 and 0.513 while specificities were 0.890, 0.884, 0.928, 0.809, 0.975, 0.865 and 0.994 respectively.

**Conclusions:**

The accuracy of ultrasound in diagnosis of PAS among women with low lying or placenta previa with previous cesarean section scars is high and recommended in all suspected cases.

**Trial registration:**

Number CRD42021267501.

**Supplementary Information:**

The online version contains supplementary material available at 10.1186/s12884-023-05675-6.

## Introduction

Placenta accreta spectrum (PAS) or previously referred to as morbidly adherent placenta, is the pathological adherence of the placenta as a result of focal or diffuse abnormal trophoblast invasion into the myometrium [1]. The rate of PAS is increasing over time. It was reported as between 1 in 2,510 and 1 in 4,017 in 1970s and 1980s and reached 1 in 533 between1982 to 2002 [2].

This increasing rate is linked to increased rate of cesarean delivery (CD), a well-known risk factor for PAS [3]. The main risk factors for PAS are previous CD and placenta previa. In women with placenta previa, the risk of PAS is 3%, 11%, 40%, 61%, and 67%, after 1,2,3,4,5 or more CD [4].

Other risk factors include advanced maternal age, high parity, prior uterine surgeries or curettage, and Asherman syndrome [3].

PAS is associated with high maternal morbidity resulting from severe life-threatening hemorrhage, that requires blood transfusion and additional surgical interventions including hysterectomy at the time of delivery or during the postpartum period. PAS is associated with prolonged hospital stay and more ICU admissions [5].

Antenatal diagnosis of PAS is highly needed to optimize maternal outcomes and arrange the delivery at level III or IV maternal care facility [6]. Ultrasound and magnetic resonance imaging (MRI) development enabled the antenatal diagnosis of PAS [7]. Although ultrasonographic features of PAS may be seen as early as the first trimester; most women are diagnosed during their 2nd or 3rd trimesters [3]. Gray scale criteria include multiple placental vascular lacunae within the, loss of retroplacental clear zone, myometrial thinning (less than 1 mm), interruption of the serosa–bladder interface, and placental extension into myometrium, serosa, or bladder [8]. The accuracy of ultrasound varies among different studies. Some yielded 100 accuracy [9] while others reported much lower values [10].

### Objective

This systematic review and meta-analysis aimed to assess the diagnostic accuracy of 2D and 3D ultrasound in cases with PAS.

## Methods

A prospectively prepared protocol that follows the Preferred Reporting Items for Systematic reviews and Meta-Analyses (PRISMA) guidelines for meta-analysis was registered at PROSPERO The registration number was CRD42021267501.

### Eligibility criteria, information sources, search strategy

Two authors (AM, NK) searched MEDLINE, CENTRAL, EMBASE, Web of Science, the Cochrane Central Register of Controlled Trials electronic databases from inception to February 2022 using the keywords related to placenta accreta, increta, percreta, morbidly adherent placenta, placenta accrete spectrum, obstetric ultrasound and their MeSH terms. Abstracts of obstetric conferences, google scholar and reference lists of the subject related studies were checked for any additional studies.

Contacting the authors was done if any clarifications or additional data were needed through emails.

### Study selection

All available trials that involved prenatal diagnosis of PAS using 2D or 3D ultrasound with subsequent pathological confirmation of PAS postnatal were carefully evaluated. All types of studies whether cohort, case control or cross sectional were included. All women with low lying placenta or placenta previa whether anterior or posterior were included. Our systematic review also included studies evaluating imaging accuracy during the 2nd or 3rd trimesters.

The primary outcomes of our review were the sensitivity and specificity of 2D ultrasound in diagnosis of PAS. Other outcomes included the accuracy of 3D ultrasound in diagnosis of PAS, scoring systems used to evaluate the condition and accuracy of ultrasound in diagnosis of the severity and depth of invasion of PAS.

### Data extraction

Data extraction of all identified studies was done by two investigators (AM and NK) after their assessment. Data extraction was independently done and any arguments were reviewed by other coauthors and if needed by authors contact. Extracted data included locations, type of the study, participants number and characteristics, Type and position of placenta previa, Ultrasound type, timing and technique (2D, and 3D),PAS grade and severity, overall accuracy, the numbers of true positive, false positive, true negative, false negative, sensitivity and specificity and sensitivity and specificity of individual ultrasound markers.

### Assessment of risk of bias

Quality assessment of the included studies was done following the Newcastle–Ottawa Scale (NOS) [11] for cohort and case control studies by two investigators (AM and NK) and disagreements were discussed further with other investigators. Missed and unclear data were checked by contacting the authors via emails and consultation of experts [11].

The GRADE system was used to assess the quality of evidence. GRADE included risk of bias of the included studies, inconsistency, indirectness, imprecision, and publication bias. Each item decreases the evidence by 1 level if have serious concerns and by 2 levels if have very serious concerns. The levels were high (if we are very confident that the true effect is close to the effect estimate), moderate (if we are moderately confident that the true effect is close to the effect estimate, but there is a possibility of a substantial difference), Low (if we have limited confidence that the true effect is close to the effect estimate and the true effect may be substantially different from the effect estimate) or very low (if we have very limited confidence in the effect estimate and the true effect is likely to be substantially different from the effect estimate).

### Data synthesis

The heterogeneity of studies included was evaluated by *I*^2^ statistic and Cochran’s Q test. Random effect model was used to calculate the overall and individual parameters diagnostic accuracy of MRI for the diagnosis of PAS. Calculated data included pooled sensitivity, specificity, positive and negative likelihood ratio. The summary receiver operating characteristic (SROC) curve and the area under the curve (AUC) were created using a regression model. Statistical difference was defined as *p* < 0.05. Forest plots were obtained to present the results graphically using open meta-analyst 12.11.14. Review Manager [RevMan] version 5.4.1 [The Nordic Cochrane Centre, Cochrane Collaboration, 2020, Copenhagen, Denmark] was used to create tables and Prisma flow chart.

## Results

### Study selection

**Fig. 1:**
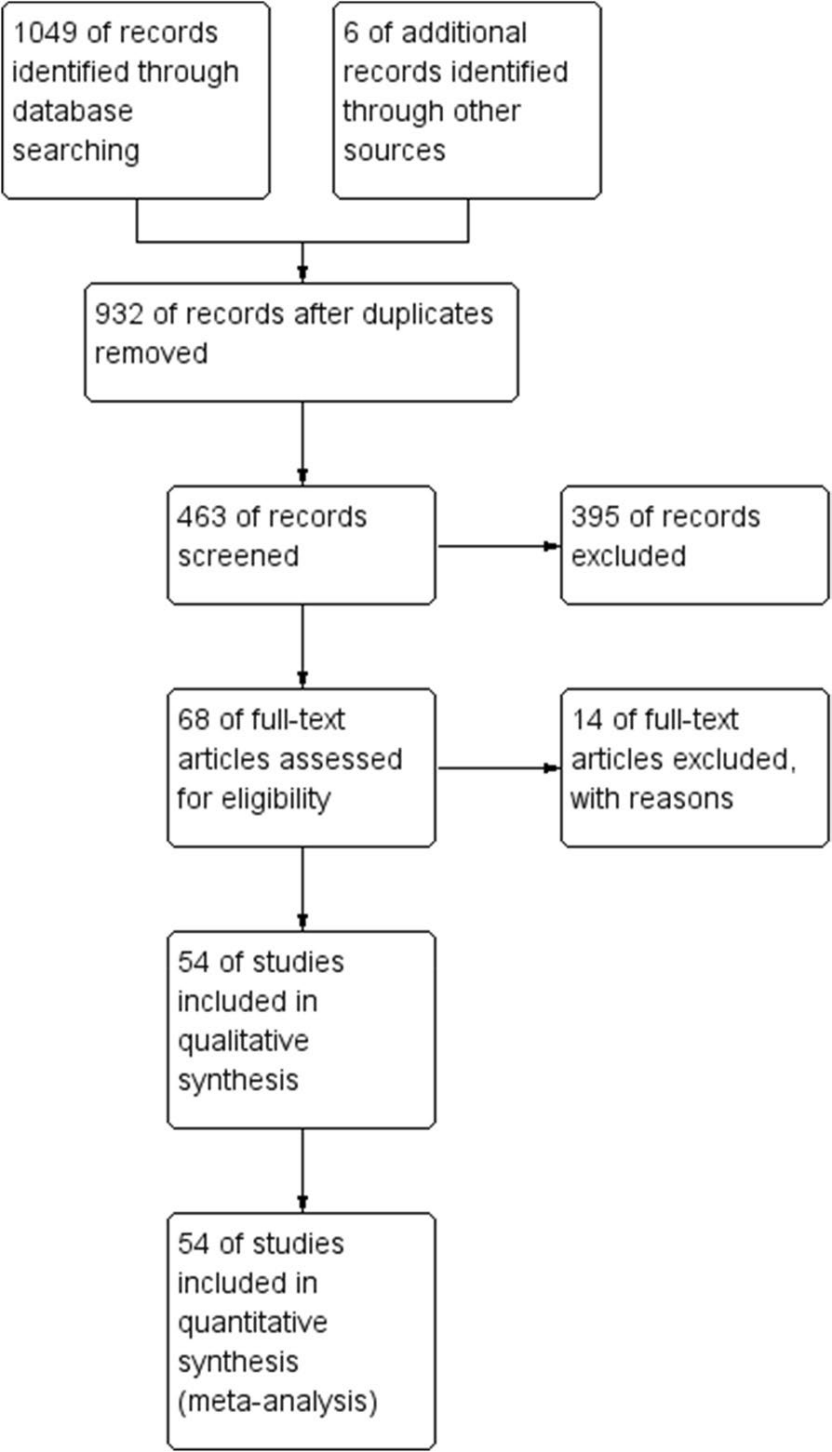
Study flow diagram

Our search yielded 1049 studies through databases and 6 records identified through other resources, 463 of them were screened after removal of duplicates, 68 studies were considered for inclusion and finally 54 studies were included (Fig. 1). The causes of exclusion were non reporting or unclear reporting of prenatal diagnosis.

### Study characteristics

Fifty-four studies included 5307 women fulfilled the inclusion criteria, PAS was confirmed in 2025 of them. Two studies were multicenter [12, 13], 3 studies were conducted in 3 centers [14–16], 1 study in 2 centers [17], 6 studies had unknown numbers of centers [18–23]and the rest 42 studies were single center. All the studies were written in English except two. One was available in French [24]and 1 was available in Portuguese [25].

The included studies were prospectively conducted in 32 studies [9, 13–15, 18–22, 26–48] and retrospectively conducted in 17 studies [16, 17, 23–25, 49–58]. Two studies were a collection of prospective and retrospective design [12, 59], and 3 studies were cross sectional [10, 60, 61].

Fourteen and eleven studies were conducted in USA [16, 21, 33, 38, 50–56, 58, 62, 63] and Egypt [9, 10, 26, 28, 32, 34, 42, 47, 48, 59, 64] respectively. The other 29 studies were conducted as follow: 6 in China [18, 23, 30, 37, 43, 65], 5 in Italy [20, 22, 35, 46, 57], 2 in France [17, 24], 2 in India [39, 61], 2 in Iran [14, 60], 2 in Turkey [31, 45], 1 in each of the following Brazil [25], Jordan [49], Japan [36], KSA [51], Malaysia [15], Pakistan [44], Qatar [27], Taiwan/Argentina [19], UK [13] and the last was conducted in 15 countries [12].

All ultrasound diagnoses were done during the 3rd trimester. In 4 studies diagnosis was done during both 2nd and 3rd trimesters of pregnancy [20, 23, 33, 62]. Three dimensional ultrasound were used in 3 studies [9, 19, 20]. The location of placenta was anterior in 5 studies [10, 20, 21, 26, 63], posterior in 3 studies [25, 35, 46] and in 46 studies the placental location included both anterior and posterior positions. Nine studies used different scoring systems in prediction of PAS [14, 26, 30, 35, 43, 46, 53, 55, 65]. In 8 studies the depth of invasion was evaluated [9, 12, 26, 43, 50, 54, 59, 66].

Table S1 summarized the main characteristics of the included studies.

### Risk of bias of included studies

Quality assessment of the included studies using Newcastle–Ottawa Scale is shown in Table 1

**Table 1:** Quality assessment of the included studies using Newcastle–Ottawa Scale

	Selection	Comparability	Outcome /Exposure
Abu-Hashim 2022 [26]	**	*	**
Al-Alfy 2021 [9]	***	*	**
Alchalabi 2017 [49]	***	**	**
Algebally 2014 [66]	***	*	**
Ayati 2017 [60]	***	*	**
Ballcacer 2016	**	*	**
Bassetty 2021 [61]	**	–––	**
Borg 2018 [28]	***	*	**
Bowman 2014 [51]	**	*	**
Budorick 2016	**	*	**
Cali 2013 [20]	***	*	**
Chong 2018 [30]	***	*	**
Chou 2000 [65]	**	*	**
Comstock 2004 [62]	**	––––-	**
Davutoglu 2018 [31]	***	*	*
De Marcillac 2016 [24]	***	*	*
Dwyer 2008 [16]	**	*	**
El-Wakeel 2018 [59]	***	*	**
ElHawary 2013 [32]	**	*	**
Finberg 1992 [33]	**	–––––	*
Fishman 2011 [63]	**	*	**
Fitzpatrick 2013	**	*	**
Garofalo 2019 [35]	***	*	**
Guo 2021 [37]	***	*	**
Hamada 2011 [36]	****	**	***
Hamisa 2015 [34]	*	*	*
Japaraj 2007 [15]	**	–––––	*
Knight 2018 [53]	***	*	**
Kumar 2016	***	*	**
Lerner 1995 [38]	*	*	*
Lim 2011 [67]	**	*	**
Lopes 2019 [25]	***	*	**
Luo 2019 [43]	**	*	**
Maged 2018 [10]	***	*	**
Magied 2018 [64]	***	*	**
Maher 2013 [41]	***	**	***
Mansour 2011 [42]	**	*	**
Marsoosi 2018	***	*	**
Masselli 2008 [22]	**	*	**
Morel 2020	***	*	**
Nawab 2017	***	*	**
Peker 2013 [45]	***	*	*
Pillioni 2016 [46]	***	**	***
Rac 2014	**	*	**
Rekawek 2021 [56]	***	*	**
Rezk 2014	***	**	***
Riteau 2014 [17]	**	*	**
Romero 2021	***	*	**
Shih 2009 [19]	**	*	**
Shweel 2012 [48]	**	*	**
Twickler 2000 [21]	**	*	**
Warshak 2006 [58]	***	*	**
Xia 2020 [23]	**	*	**
Zhou 2014 [18]	**	*	**

NOS 'star system' is based on three main perspectives: the selection of the study groups; the comparability of the groups; and the ascertainment of exposure (for case control) or outcome (for cohort) studies. Selection items included representative of exposed cohort, selection of non-exposed, ascertainment of exposure and whether the outcome of interest was demonstrated from the start of the study or not in cohort studies and included adequate case definition, representativeness of the cases, selection of Controls and definition of Controls in case–control studies. Comparability assessment include Comparability of cohorts (in cohort studies) or cases and controls (in case control studies) on the basis of the design or analysis. Ascertainment of the outcome in cohort studies included assessment of outcome, length of follow up and adequacy of follow up of cohorts. Assessment of exposure in case control studies included ascertainment of exposure, using the same method of ascertainment for cases and controls and non-response rate

* low risk in 1 evaluation item

**low risk in 2 evaluation item

***low risk in 3 evaluation item

****low risk in 4 evaluation item

GRADE quality of evidence for each ultrasound criteria is summarized in supplementary table S2.

### Synthesis of results

**Fig. 2:**
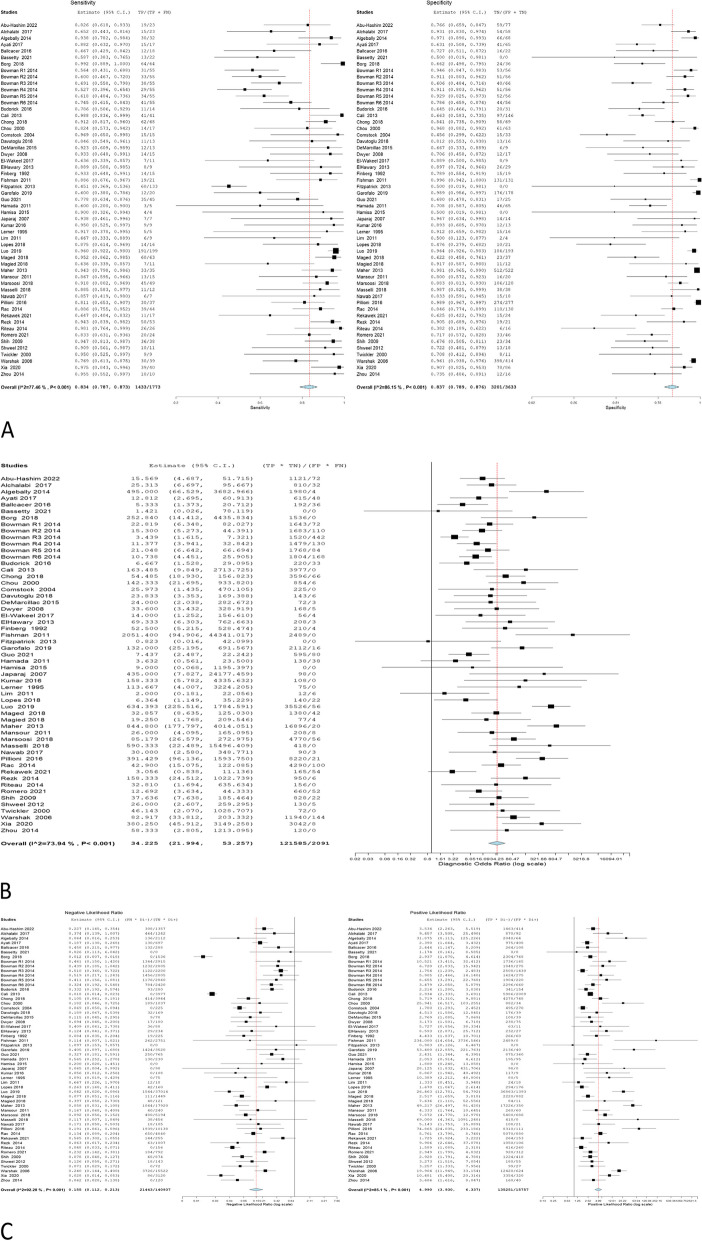
Overall **A** sensitivity and specificity, **B** Odd ratio, **C** NLR and PLR of 2D ultrasound

The accuracy of 2D ultrasound was reported in 50 studies that involved 5406 women, and 1773 of them were confirmed to have PAS through pathological examination. The overall sensitivity was 0.8703 (0.825 to 0.9051); the specificity was 0.8634 (0.8142 to 0.9012), with a -0.2348 negative correlation between them. The estimates of the Odd ratio, negative likelihood ratio, and positive likelihood ratio were 34.225 (21.994–53.257), 0.155 (0.112–0.213), and 4.990 (3.930–6.337) respectively (Table 2, Fig. 2).

**Table 2:** Ultrasonographic criteria of the included studies

		Estimate	*P* value
Overall estimate	Sensitivity	0.8703 (0.825—0.9051)	< 0.001
	Specificity	0.8634 (0.8142—0.9012)	< 0.001
	Correlation	-0.2348	
	Odds Ratio	34.225 (21.994—53.257)	< 0.001
	Negative Likelihood Ratio	0.155 (0.112—0.213)	< 0.001
	Positive Likelihood Ratio	4.990 (3.930—6.337)	< 0.001
Myometrial thinning	Sensitivity	0.7627 (0.6249—0.8611)	< 0.001
	Specificity	0.8904 (0.7154—0.9633)	< 0.001
	Correlation	-0.4938	
	Odds Ratio	13.915 (4.920—39.356)	< 0.001
	Negative Likelihood Ratio	0.284 (0.149—0.543)	< 0.001
	Positive Likelihood Ratio	3.432 (2.039—5.777)	< 0.001
Loss of retroplacental clear zone	Sensitivity	0.7799 (0.6905—0.8492)	< 0.001
	Specificity	0.8839 (0.7933—0.9379)	< 0.001
	Correlation	-0.1416	
	Odds Ratio	16.892 (7.908—36.082)	< 0.001
	Negative Likelihood Ratio	0.272 (0.188—0.394)	< 0.001
	Positive Likelihood Ratio	4.149 (2.703—6.369)	< 0.001
Bridging vessels	Sensitivity	0.6593 (0.5418—0.76)	0.009
	Specificity	0.9279 (0.7858—0.9783)	< 0.001
	Correlation	-0.5786	
	Odds Ratio	13.553 (5.520—33.277)	< 0.001
	Negative Likelihood Ratio	0.337 (0.154—0.739)	0.007
	Positive Likelihood Ratio	3.489 (2.214—5.497)	< 0.001
Placental lacunae	Sensitivity	0.7845 (0.7057—0.8468)	< 0.001
	Specificity	0.8086 (0.729—0.869)	< 0.001
	Correlation	-0.232	
	Odds Ratio	11.115 (6.826—18.096)	< 0.001
	Negative Likelihood Ratio	0.275 (0.198—0.382)	< 0.001
	Positive Likelihood Ratio	2.972 (2.288—3.860)	< 0.001
Bladder wall interruption	Sensitivity	0.4551 (0.3262—0.5902)	0.482
	Specificity	0.9747 (0.922—0.9921)	< 0.001
	Correlation	-0.2586	
	Odds Ratio	10.681 (3.393—33.622)	< 0.001
	Negative Likelihood Ratio	0.542 (0.362—0.811)	0.003
	Positive Likelihood Ratio	4.965 (2.237—11.021)	< 0.001
Exophytic mass	Sensitivity	0.2182 (0.1171—0.3702)	0.001
	Specificity	0.8653 (0.3226—0.9886)	0.216
	Correlation	0.7355	
	Odds Ratio	1.184 (0.104—13.528)	0.892
	Negative Likelihood Ratio	1.551 (0.439—5.483)	0.496
	Positive Likelihood Ratio	1.456 (0.318—6.663)	0.629
Uterovesical vascularity	Sensitivity	0.5135 (0.3051—0.7173)	0.845
	Specificity	0.9937 (0.9104—0.9996)	< 0.001
	Correlation	-0.4241	
	Odds Ratio	12.778 (4.797—34.035)	< 0.001
	Negative Likelihood Ratio	0.438 (0.256—0.748)	0.003
	Positive Likelihood Ratio	3.744 (2.074—6.759)	< 0.001

The overall sensitivity, specificity, OR, NLR, and PLR of individual ultrasonographic criteria are shown in Table 2 and Figs. 2, 3, 4, 5, 6, 7, 8, 9, 10.

**Fig. 3:**
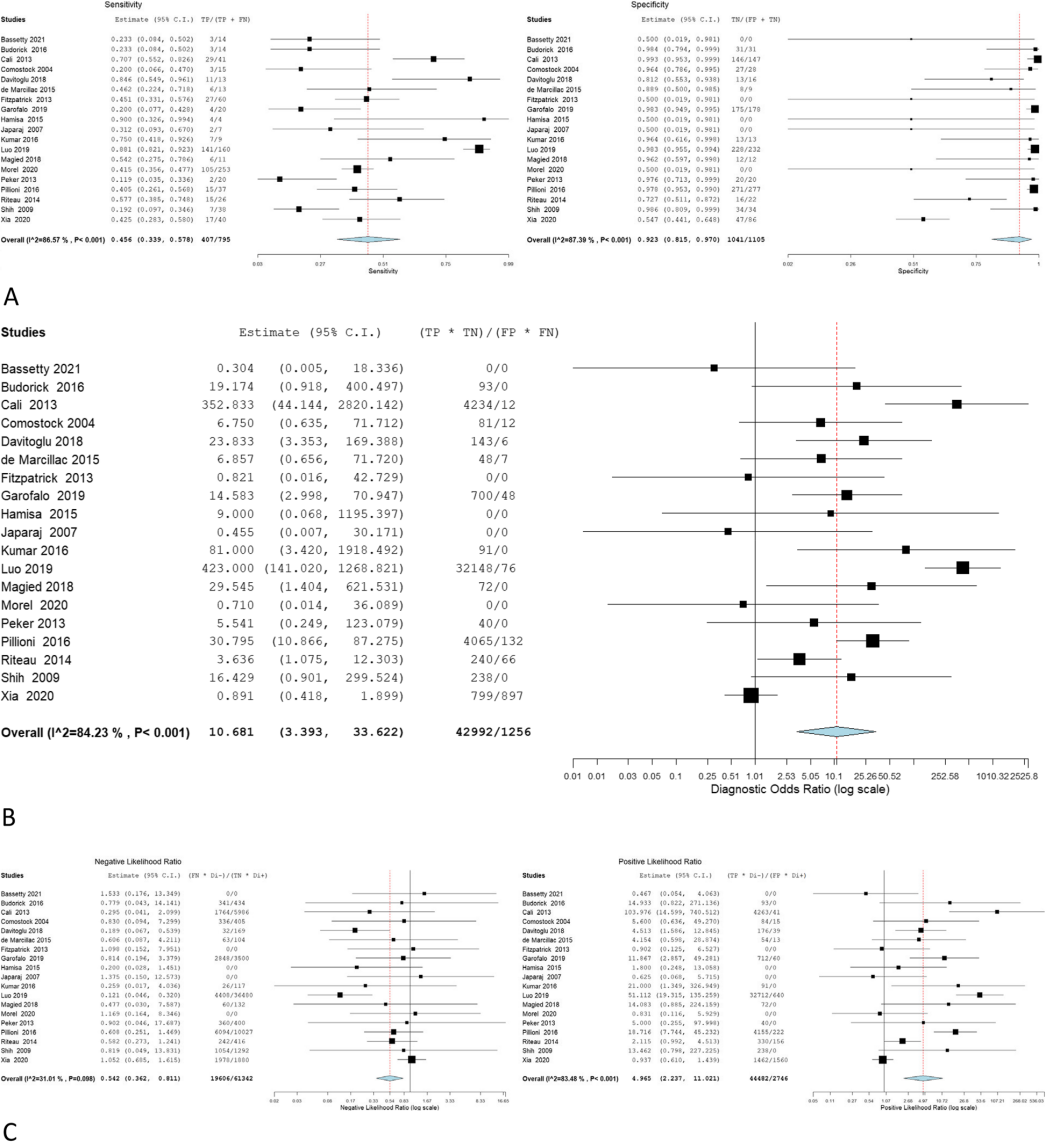
Bladder wall interruption **A** sensitivity and specificity, **B** Odd ratio, **C** NLR and PLR

**Fig. 4:**
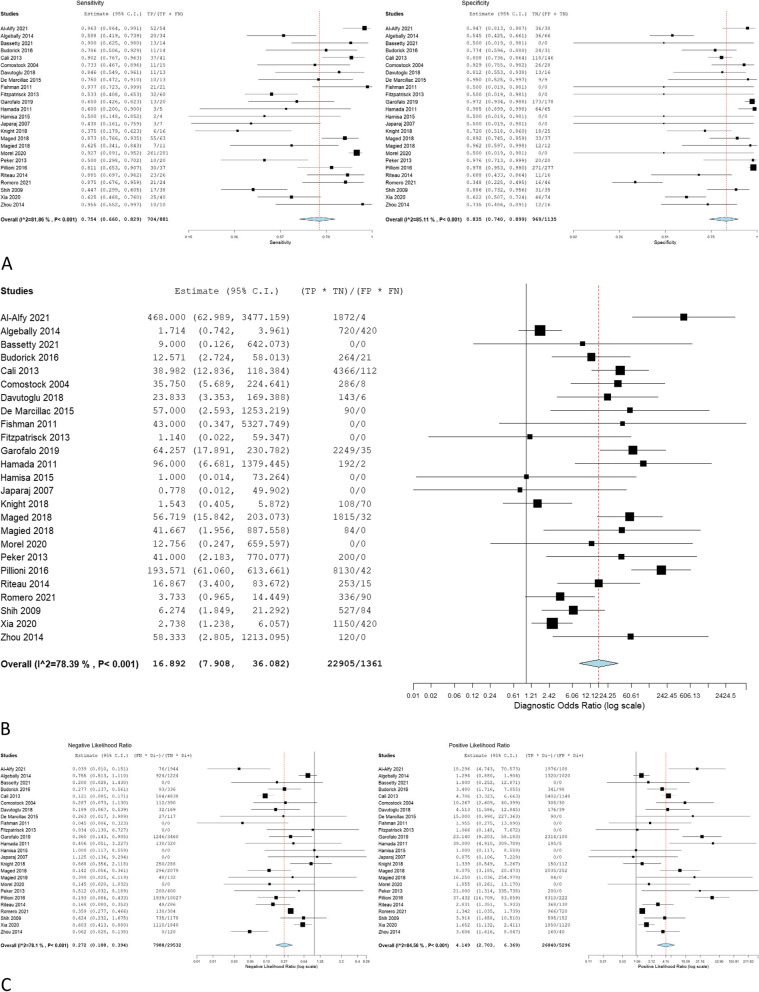
Loss of retroplacental clear zone **A** sensitivity and specificity, **B** Odd ratio, **C** NLR and PLR

**Fig. 5:**
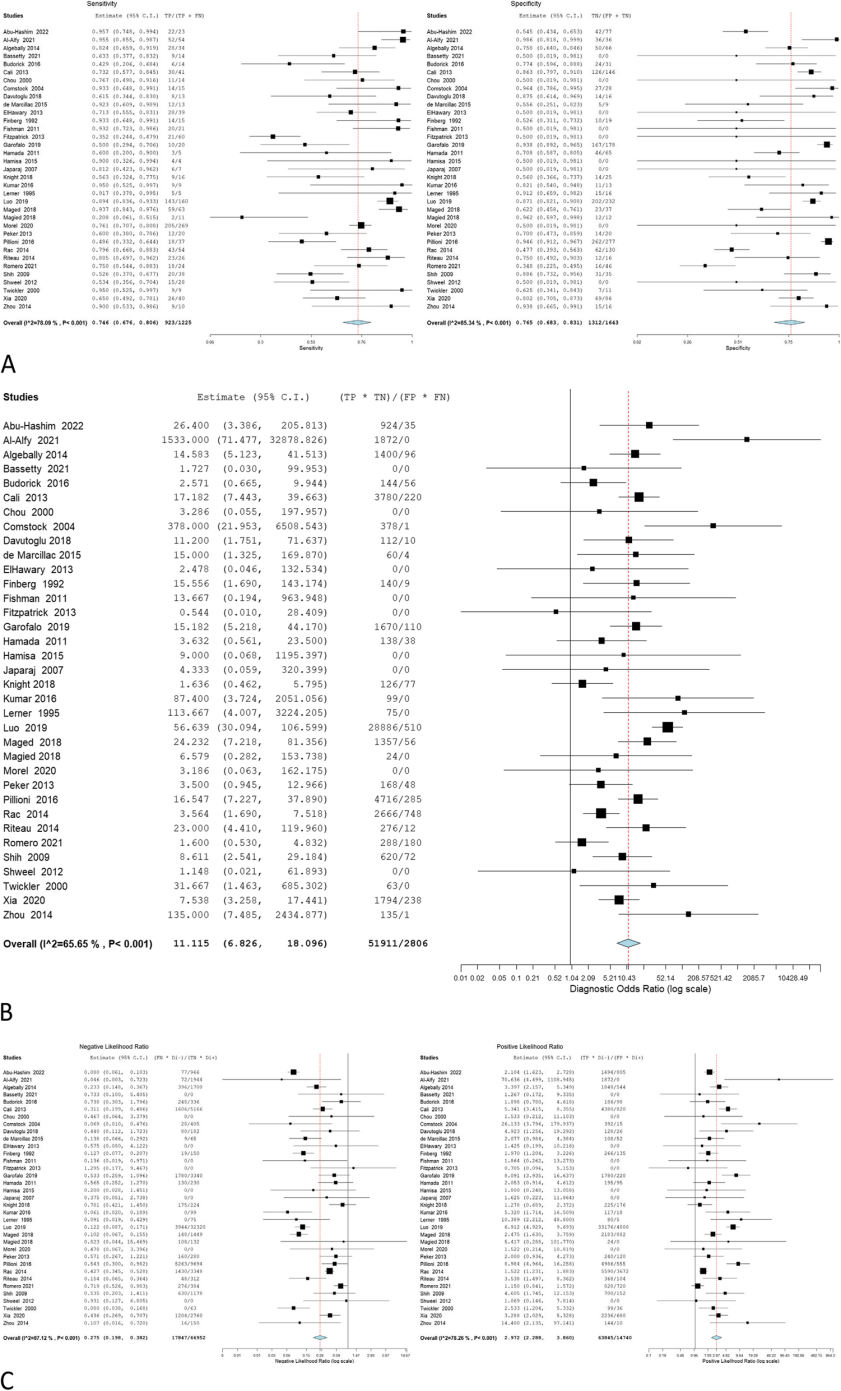
Placental Lacunae **A** sensitivity and specificity, **B** Odd ratio, **C** NLR and PLR

**Fig. 6:**
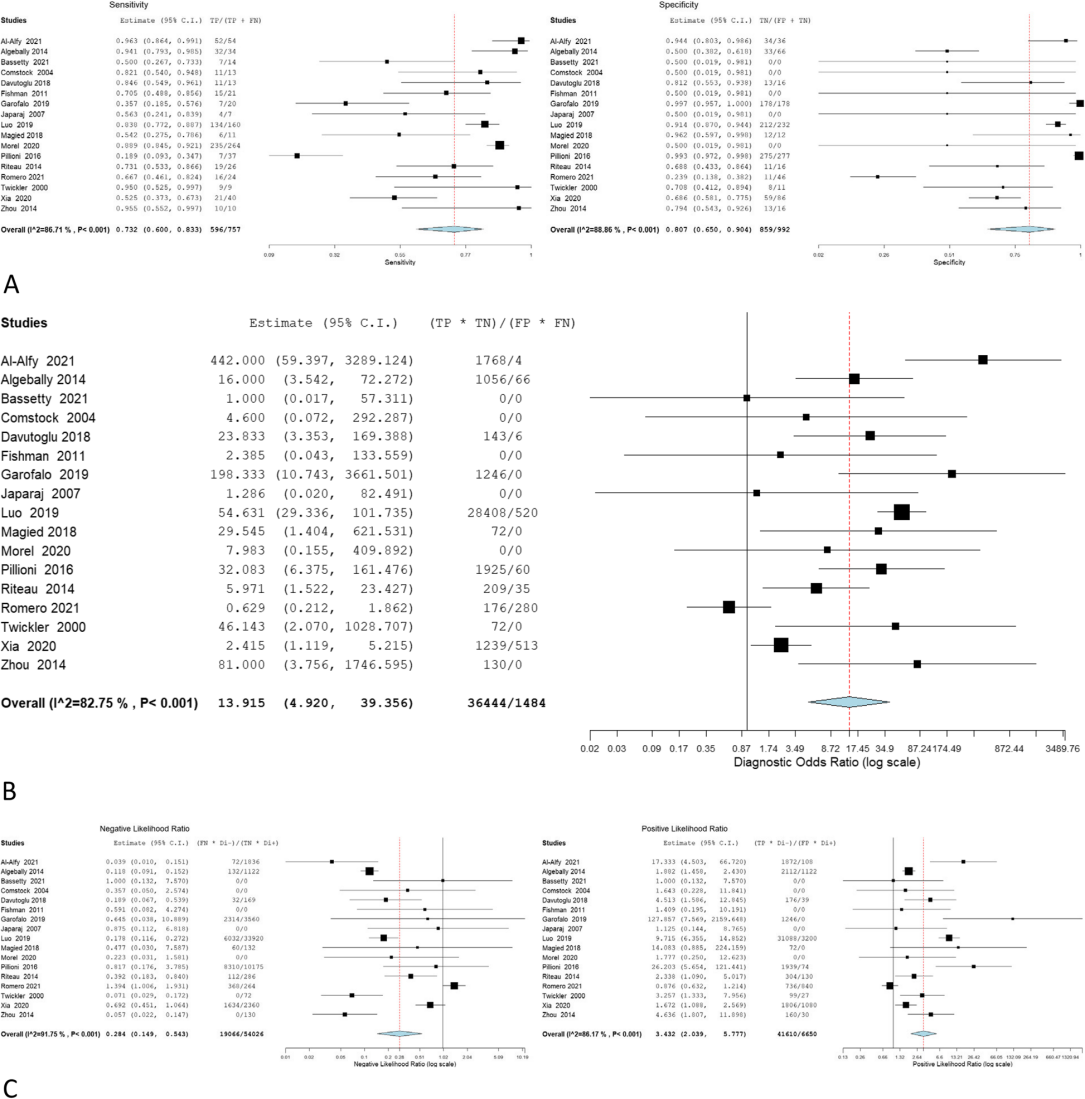
Myometrial thinning **A** sensitivity and specificity, **B** Odd ratio, **C** NLR and PLR

**Fig. 7:**
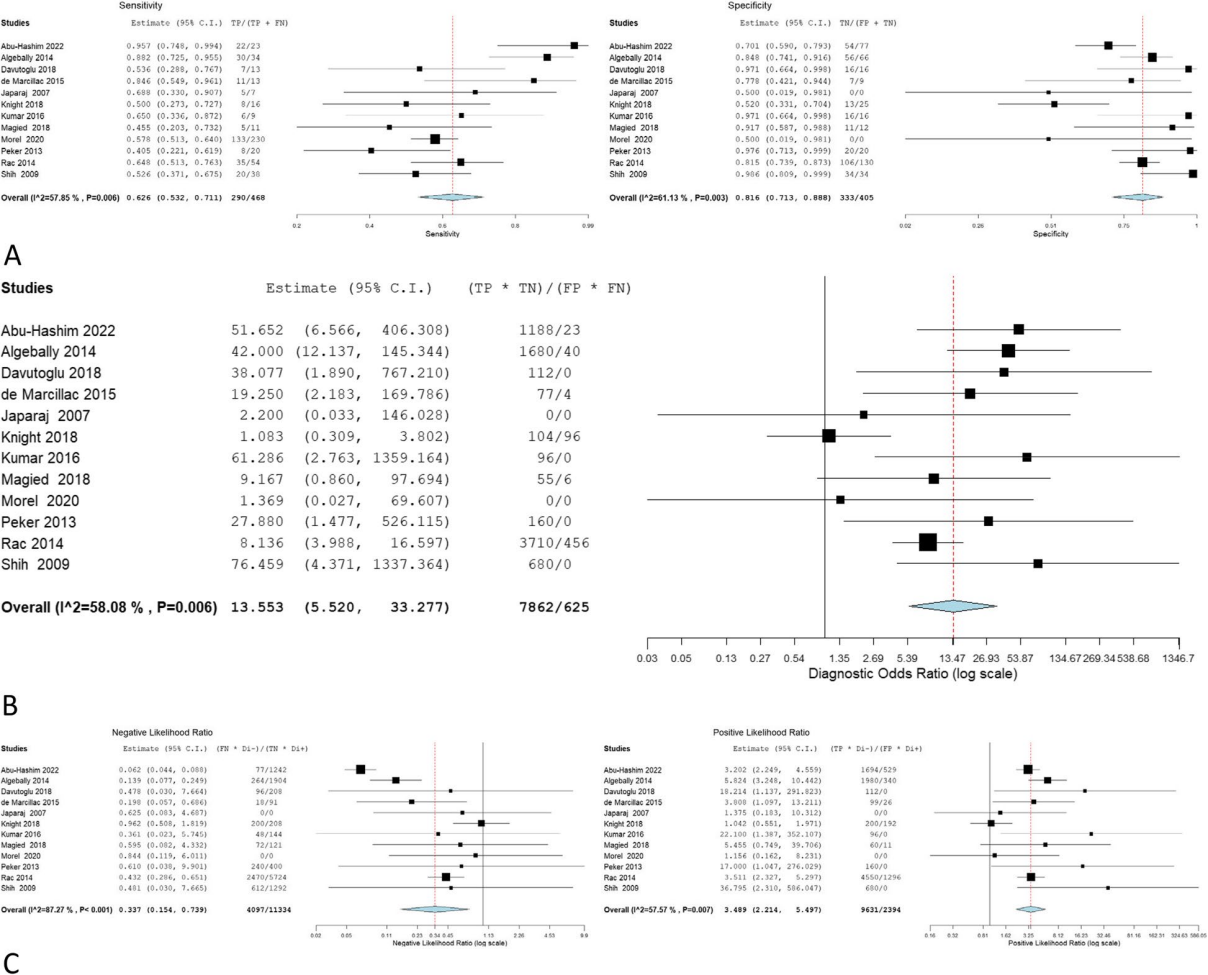
Bridging vessels **A** sensitivity and specificity, **B** Odd ratio, **C** NLR and PLR

**Fig. 8:**
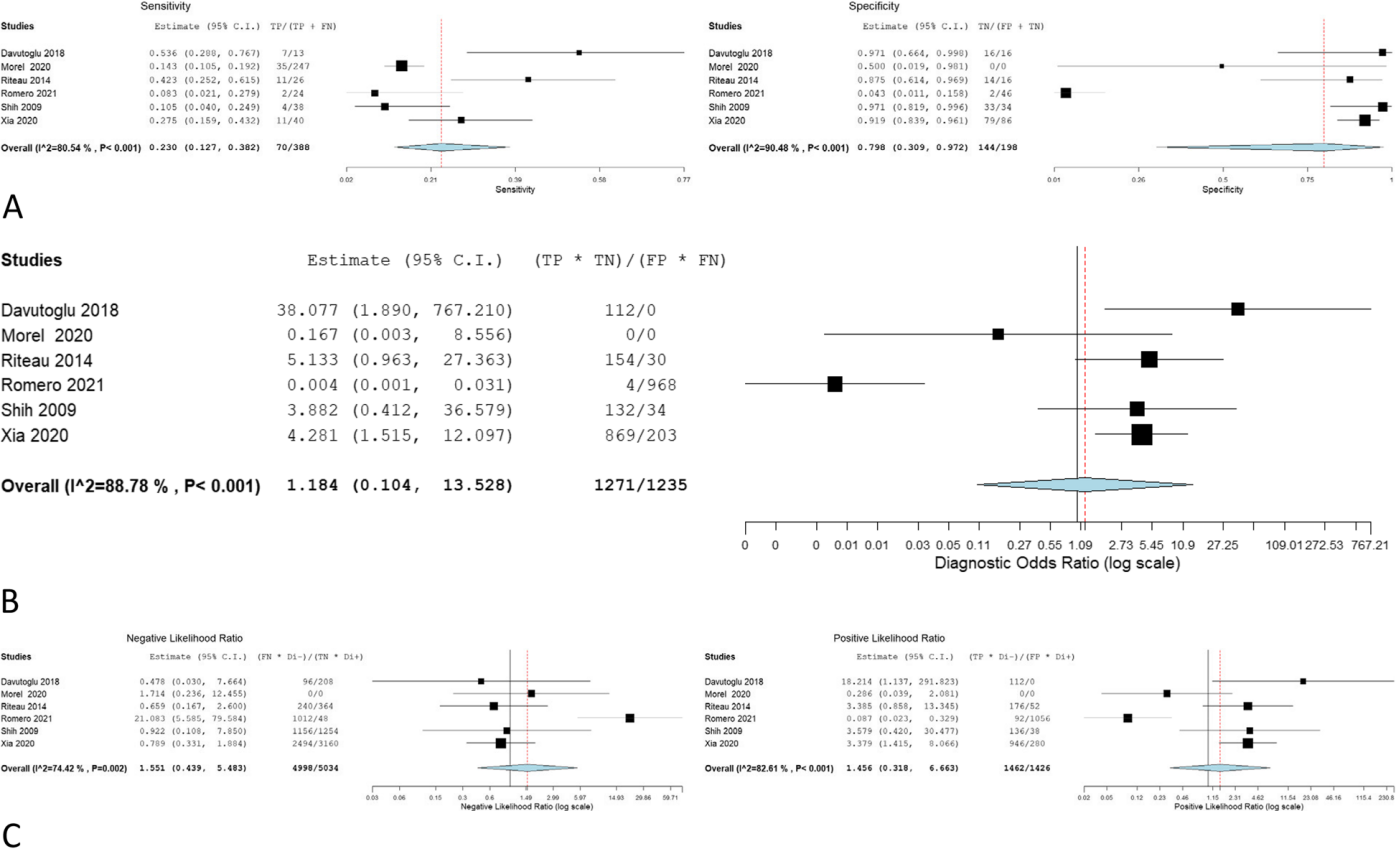
Placental exophytic mass **A** sensitivity and specificity, **B** Odd ratio, **C** NLR and PLR

**Fig. 9:**
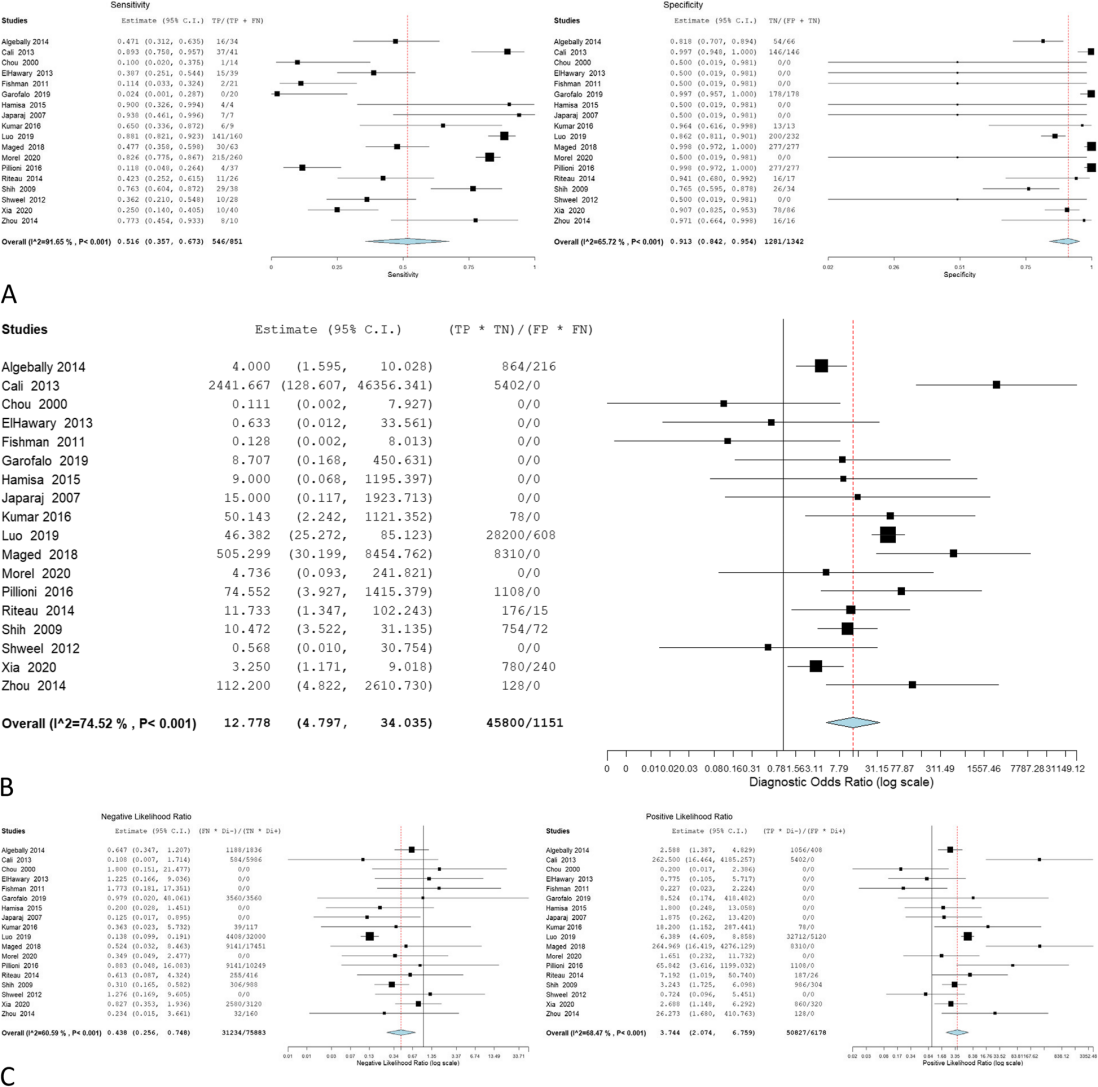
Uterovesical vascularity **A** sensitivity and specificity, **B** Odd ratio, **C** NLR and PLR

**Fig. 10:**
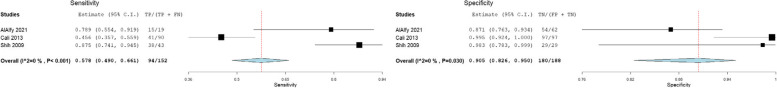
Overall **A**: sensitivity and specificity, 3D ultrasound

The accuracy of 3D ultrasound was reported in 3 studies that involved 447 women, and 134 of them had a confirmed diagnosis of PAS. The overall estimates of sensitivity and specificity were 0.728 (0.379–0.921) and 0.969 (0.764–0.997), respectively (Fig. 10).

**Fig. 11:**
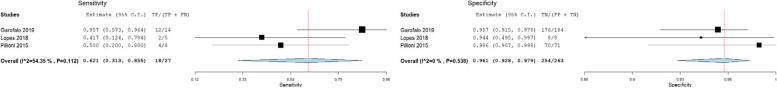
Overall **A**: sensitivity and specificity of Posterior placenta

The accuracy of ultrasound in posterior PAS was reported in 3 studies that involved 290 women, 27 of whom had a confirmed diagnosis of PAS. The overall estimates of sensitivity and specificity were 0.621 (0.313–0.855, P value 0.450) and 0.961 (0.928–0.979, *P* value < 0.001), respectively (Fig. 11).

Supplementary Figs. (1–10) describe ROC curves for all individual criterion.

Ultrasound was used in eight studies to assess the depth of invasion in PAS. One study [26] used the placenta accreta index score.

Alalfy and colleagues [9] used a simplified 3D ultrasonographic criteria that identified all 16 cases of placenta accreta, 23 /24 cases with increta (1 was diagnosed as accreta) and all 14 cases of placenta percreta. These criteria were able to identify all 28 cases of focal invasion and missed one out of 26 cases of diffuse placental invasion (mistaken as focal invasion). They reported an accuracy of 98.8 in detection of PAS severity. Algebally et al. [27] reported a false negative diagnosis in 4 cases of accreta (12 cases), 4 cases of increta (8 cases) and a false negative diagnosis of 8 cases of percreta (12 cases) while all non-adherent cases were accurately detected.

Balcacer and colleagues [50] reported that ultrasound was able to detect accreta/increta versus percreta with 71% accuracy in 12 of 17 women with PAS. In one study [65], all women with PAS (8 accreta, 6 increta, and no percreta) met at least one of the sonographic criteria (accuracy 100%).

The frequency of ultrasonographic criteria in 105 women diagnosed as Accreta and/or Increta and 213 diagnosed as Percreta were as follows: loss of clear zone (96.2% vs. 86.6%), myometrial thinning (96.7% vs. 72.9%), bladder wall interruption (52.2% vs. 24.0%), placental bulge (59.9% vs. 28.7%), uterovesical hypervascularity (90.8% vs. 66.7%), bridging vessels (67.3% vs. 40%), and parametrial involvement (11.8%) [12].

ElWakeel and colleagues [59] reported that loss of the retroplacental clear zone diagnosed all 3 accreta, 3 increta, and 1 percreta; interruption of the bladder wall missed 1 case of accreta; the presence of placental lacunae missed 3 cases of accreta and 2 cases of increta; the myometrial thinning criterion missed 1 case of increta; and subplacental hypervascularity missed 2 cases of accreta and 3 accreta.

Lim et al. [67] evaluated ultrasound in the diagnosis of severity of PAS in 13 cases (5 accreta, 3 increta, 1 percreta, and 4 non-adherent). They reported FN in three adherent cases; all three increta and one percreta were TP, while two of the non-adherent cases had a FP diagnosis.

In eight studies, different scoring systems were evaluated. The placenta accreta index (PAI) was assessed in 2 studies [26, 55]. This index was calculated by combining data from the placental site, the number of previous CDs, the measurement of the smallest sagittal myometrial thickness, the presence of placental lacunae, and bridging vessels. They reported a mean score of 6.91.2, 7.41.4, and 8.71.1 in accreta (8 women), increta (7 women), and percreta (8 women) respectively. AbuHashim et al. described that the 5.37 cut-off point for PAI had a sensitivity of 83.9%, a specificity of 76.3%, a PPV of 85.2%, an NPV of 74.3%, and an accuracy of 81%. PAI scores of > 0, 1, 2, 3, 4, 5, 6, 7, and 8 had invasion probabilities of 5, 10, 19, 33, 51, 69, 83, 91, and 96, sensitivity of 100, 97, 93, 86, 72, 52, 31, and 24, and specificity of 19, 47, 58, 68, 85, 92, 100, 100, and 100, respectively. Two criteria systems were evaluated in two studies [46] and [35]. Garofalo et al. reported that the two-criteria system diagnosed 12/20 women with PAS, providing a sensitivity, specificity, PPV, and NPV of 60%, 98.9%, 85.7%, and 95.7, respectively, while in the Pillioni et al. study, it diagnosed 30/37 women with PAS, providing a sensitivity, specificity, PPV, and NPV of 81.1%, 98.9% (274/277), 90.9%, and 97.5%, respectively. Scoring systems including clinical and ultrasonographic criteria were used in 3 studies [14, 30, 43]. Clinical criteria included the number of previous CDs and ultrasonographic features including placental location and other criteria, especially the presence of placental lacunae. Luo and colleagues reported a threshold score between 2.25 and 6.2 predicted placenta accreta with 80.26 PPV and 94.3 NPV, a threshold score between 6.2 and 8.95 predicted placenta increta with 75.47 PPV and 96.17 NPV, and a threshold score of 8.95 or more predicted placenta percreta with 81.81 PPV and 97.3 NPV. They reported high PPV (95.44%) and 81.81% for women without PAS and with percreta respectively, and moderate (80.26%) and 75.47% for women with accreta and increta, respectively. They reported a high NPV of 5.44%, a low FP rate of 3.32%, and without PAS, very low FP rates of 4.56%. Marsoosi et al. categorized women into low, moderate, and high probability groups. In the low, moderate, and high probability groups, 4 (3.92%), 23 (65.71%), and all patients had accreta. When AUC was 98%, the overall sensitivity, specificity, PPV, and NPV were 91.84%, 87.27%, 86.54%, and 92.31%, respectively. Chong et al. categorised patients as N1, N2, and N3 when the score was ≤ 5, 6–9, and ≥ 10. The absence of PAS was detected in 62/69 in the N1 group and in 8/58 in the N2/N3 group. The presence of percreta was detected in 3 patients in the N1/N2 group and in 28 women in the N3 group (of whom 25 had a confirmed pathological examination).

One study [53] combined ultrasonographic markers with MRI criteria in a scoring system. They assigned patients a low, intermediate, or high score.The percentage of women in normal, accreta, and increta/percreta were 90, 10, and 0% in the low category; 42.9, 28.6, and 28.6 in the intermediate category; and 14.3, 28.6, and 57.1 in the high category.

Among these different ultrasonographic and Doppler criteria, the presence of placental lacunae showed the highest sensitivity (0.7845) and the lowest specificity (0.8086). The highest specificity was detected using the presence of uterovesical hypervascularity (0.9937), while the lowest sensitivity was reported in exophytic placental mass criteria (0.2182).

## Discussion

### Main findings

The current meta-analysis included 54 prospective cohort, retrospective case control and cross sectional studies that conducted on 2025 women diagnosed pathologically with PAS compared to 3282 women high risk for PAS but don’t have such diagnosis. The sensitivity and specificity of 3rd trimester 2D ultrasound were 0.8703 and 0.8634 respectively.

The use of 3D ultrasound didn’t add too much to the value of 2D ultrasound (although it was evaluated in 3 studies only).

The accuracy of diagnosis of PAS was lower in cases with posterior location of the placenta. \(3 studies only were focused on posterior placenta).

A large number of the included studies were conducted in low- and middle-income countries as these have the higher number of cesarean sections and higher parity distribution compared to high income countries. Also, 2D ultrasound represent a low cost diagnostic tool available in governmental hospitals and private centers.

FIGO classified PAS into 3 categories and classified grade 3 to another 3 subgroups 3a, b and 3c according to clinical criteria at vaginal delivery and laparotomy and histological criteria [68]. Ultrasound and MRI are the main tools used for diagnosis of PAS. Three-dimensional ultrasound add the value of increasing the contrast between different tissues allowing better detection of placental growth within the surrounding tissues and better assessment of myometrial thickness [9].

### Strengths and limitations

Our meta-analysis is the first one focusing on the diagnostic accuracy of ultrasound of PAS in women of low-lying placenta or placenta previa overlying a uterine scar. It included the largest number of studies reached by extensive searching of all available data bases and the grey literatures, trial registration sites, reference list of all related studies. It included diagnosis using both 2D and 3D ultrasound. A separate analysis was done for those with posterior placentae and for studies that combined ultrasonographic markers with clinical evaluation. Also, a separate analysis was done for each of the diagnostic criteria to arrange the criteria regarding their importance in diagnosis and exclusion of PAS. Also, evaluation of ultrasound accuracy in diagnosis of the severity of PAS was done. The use of quality assessment was done to properly assess the risk of bias among the studies.

The heterogeneity of the included studies was the main limitation of our meta-analysis. So we used the most adequate tool of random effect for comparison. However, Meta-analysis for assessment of severity and different scoring systems was not possible due to marked heterogeneity among the studies. Most of the included studies were not registered. Some studies assessed only sensitivity and PPV as they didn’t include controls.

### Comparison with existing literature

Jauniaux and Bhid metaanalysis [68] was done in 2017 and included 14 studies and the review was not focused on diagnostic accuracy. It included evaluation of risk factors and outcomes. There was no individual analysis of ultrasonographic criteria. Since then, many new studies were conducted and marked development in ultrasound machines was achieved. Another more recent review was conducted to assess the risk factor, and diagnostic accuracy of prenatal ultrasound and MRI in detecting PAS in women with posterior location of the placenta [69]. This review included 20 study and only 11 of them evaluated the accuracy of imaging technique. Again the study was not focused on diagnostic accuracy but also evaluated risk factors. There was also no evaluation of individual ultrasonographic criteria.

So, our study provides the evidence of diagnostic accuracy of ultrasound in diagnosis of PAS and its severity. It included all the available studies with comprehensive subgroup analysis.

## Conclusions and Implications

Our systematic review confirmed the value of ultrasound in diagnosis of PAS among women having a low lying or placenta previa with previous uterine scars. Its use is mandatory as it represents a low cost, readily available tool for prenatal diagnosis of PAS. Prenatal diagnosis is highly valuable in optimization of maternal and neonatal outcome. It enables the formation of a multidisciplinary team and the best available resources, including ICU and blood transfusion preparedness. We advise integrating all sonographic markers because numerous studies have demonstrated that doing so improves sensitivity when combined with a woman's clinical features. We do recommend higher quality prospective studies that combine different sonographic criteria to be conducted to ensure the exact accuracy of the ultrasound. In order to accurately analyse the variation in accuracy across users with varied expertise, examinations that evaluate the accuracy of ultrasonography should also involve sonographers with different levels of competence.

## Supplementary Information

**Additional file 1.** Supplementary figures

**Additional file 2.** Supplementary tables

## Data Availability

All data of this systematic review are available within the text and tables within the manuscript.
