# Supplementary material for: Diagnostic accuracy of ultrasound in the diagnosis of Placenta accreta spectrum: systematic review and meta-analysis
Source: BMC Pregnancy Childbirth. 2023 May 15;23:354. doi: 10.1186/s12884-023-05675-6 (PMC10186814; doi:10.1186/s12884-023-05675-6)
Supplement: Supplementary file 1 — Additional file 1. Supplementary figures [file 12884_2023_5675_MOESM1_ESM.docx]

Supplementary figures


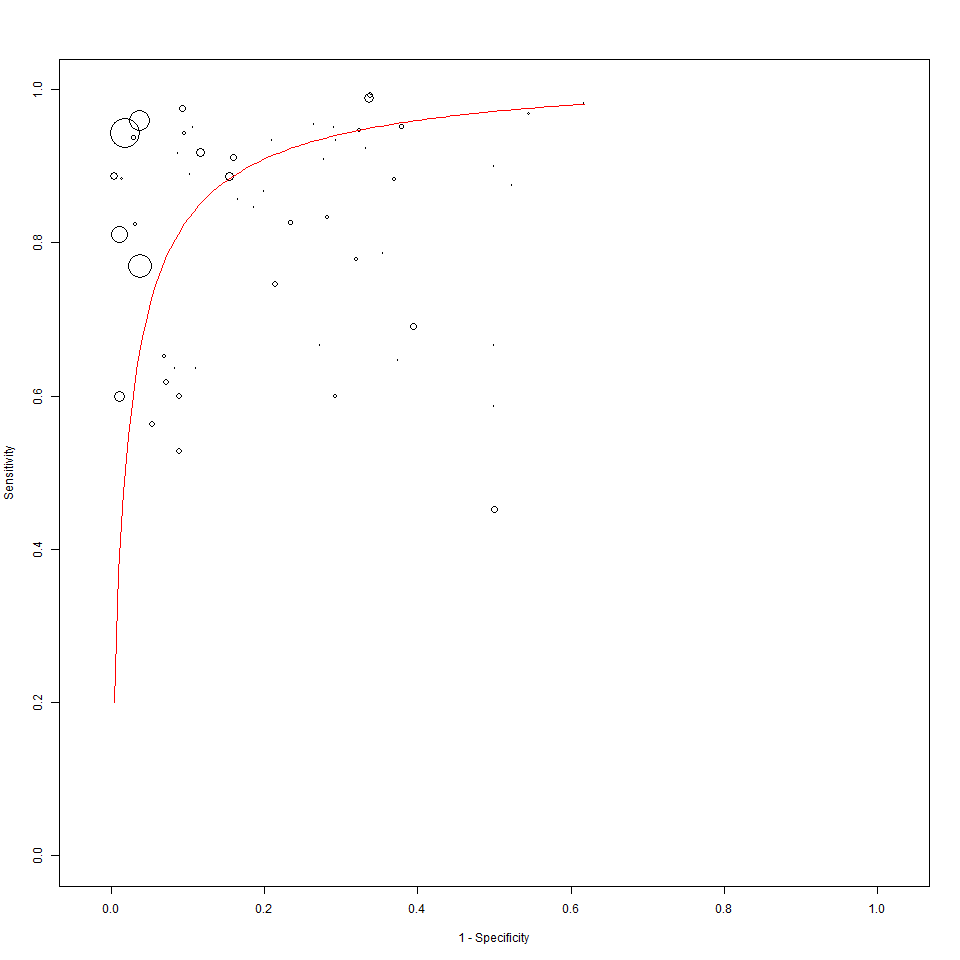


Figure S1 Overall A: ROC of 2D ultrasound


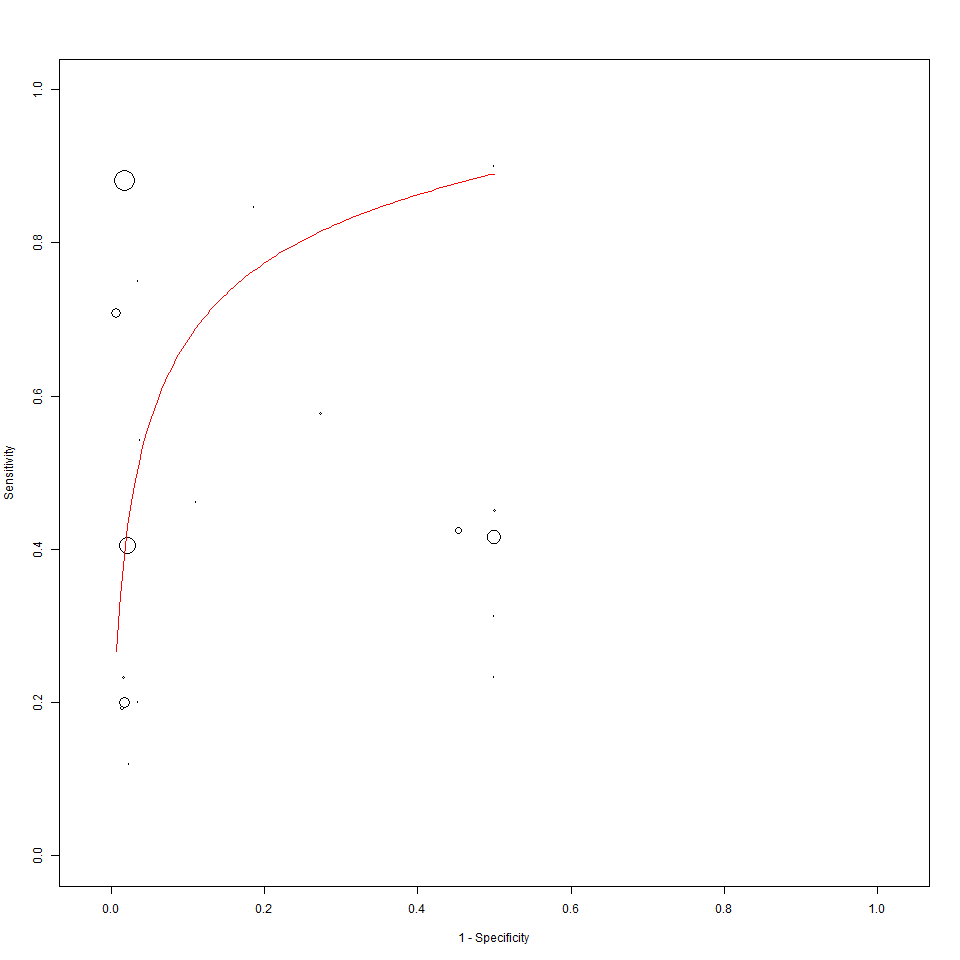


Figure S2 Bladder wall interruption A: ROC curve


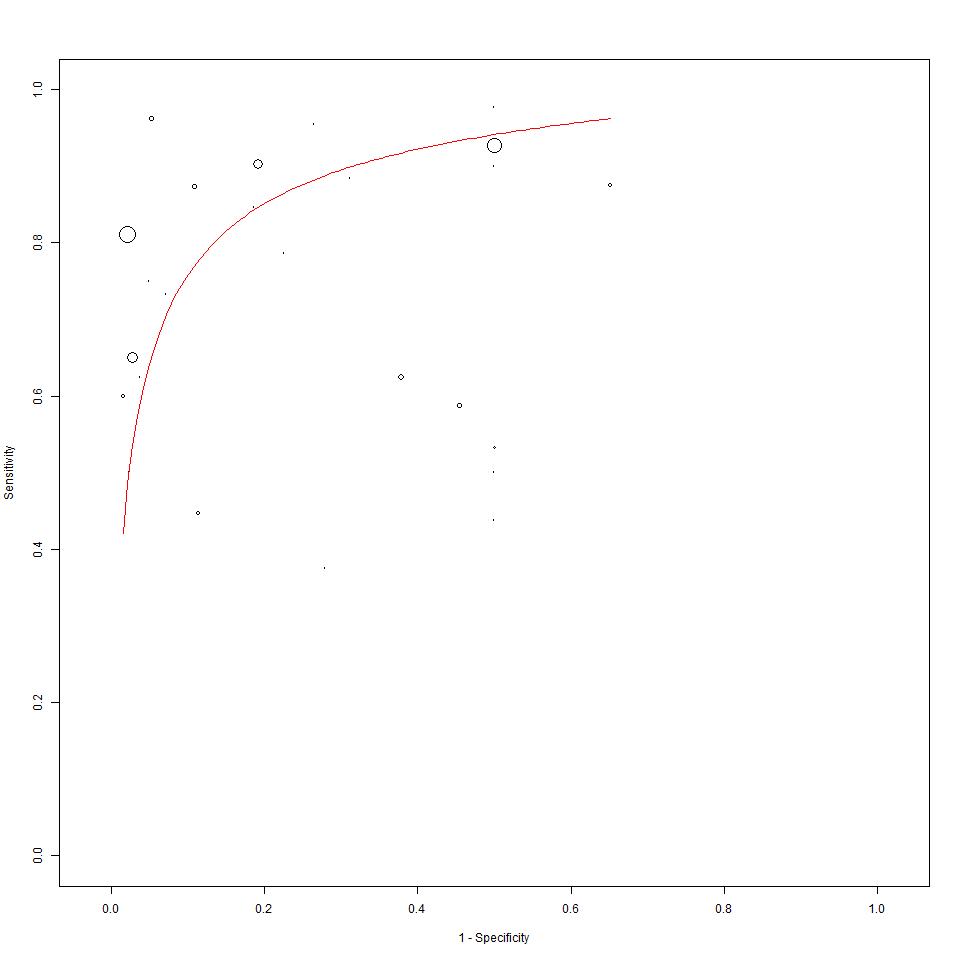


Figure S3 Loss of retroplacental clear zone A: ROC curve


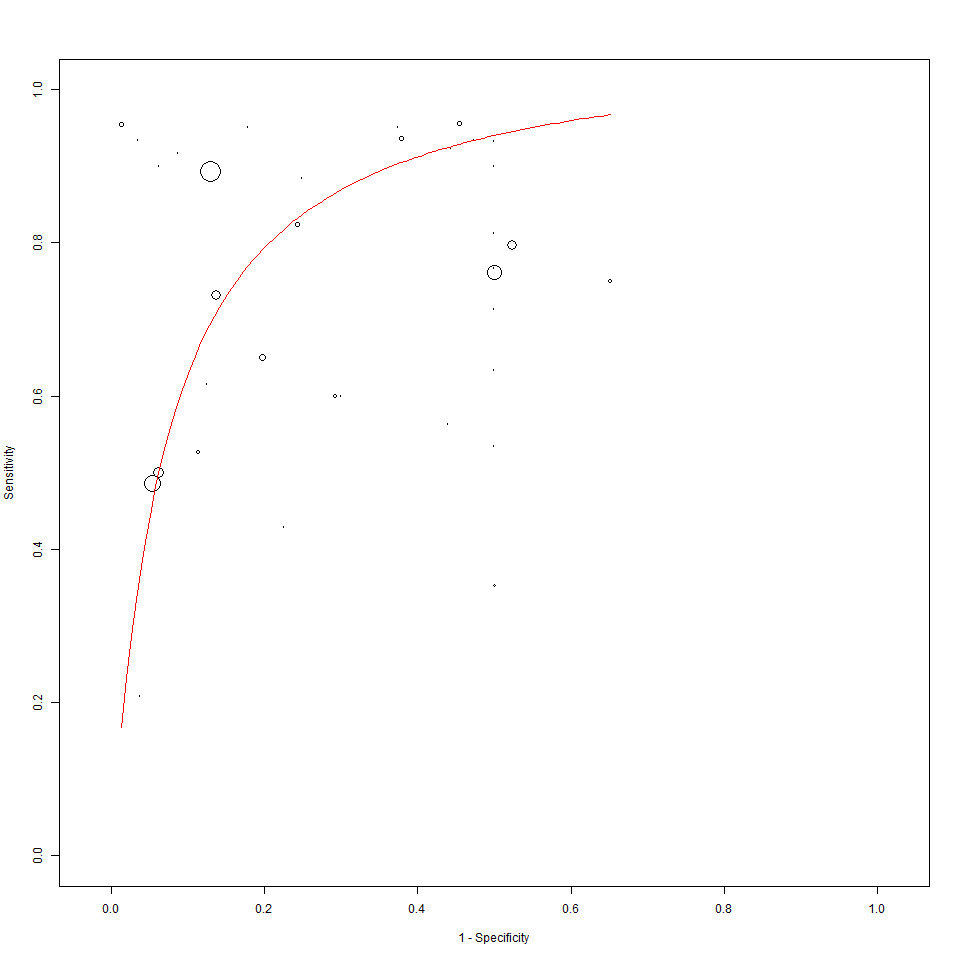


Figure S4 Placental Lacunae A: ROC curve


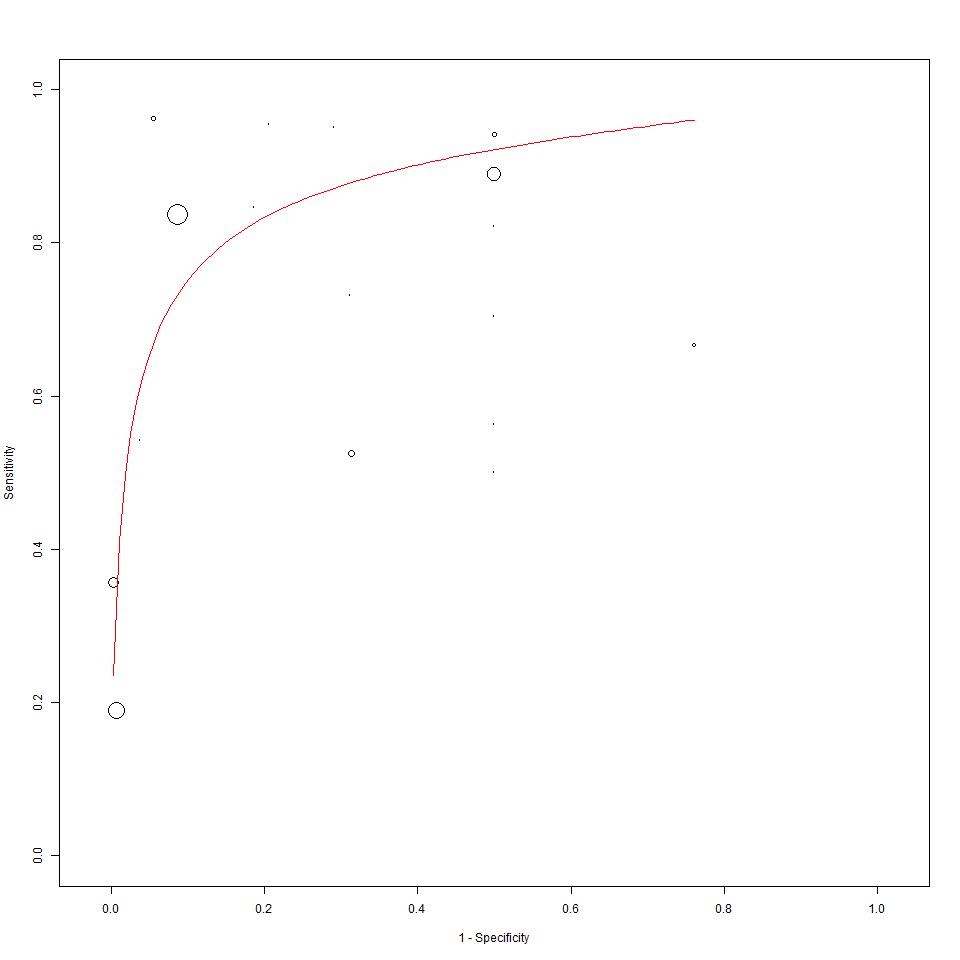


Figure S5 Myometrial thinning A: ROC curve


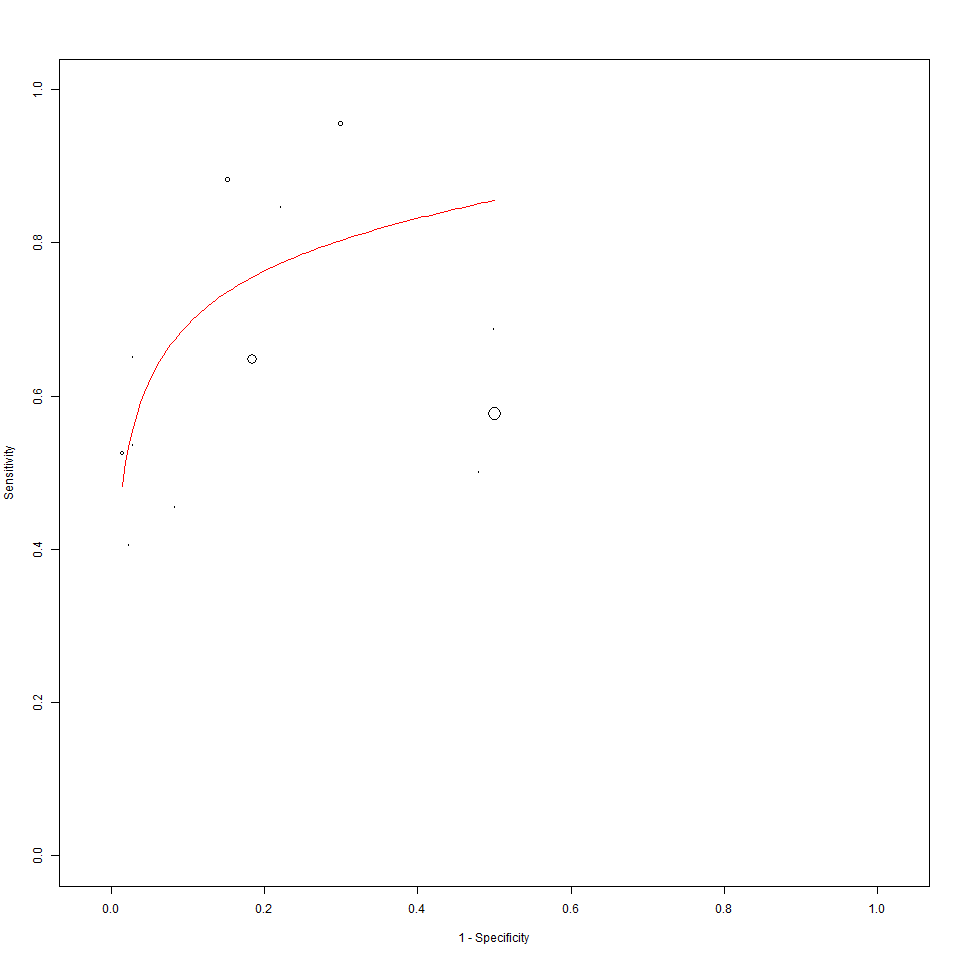


Figure S6 Bridging vessels A: ROC curve


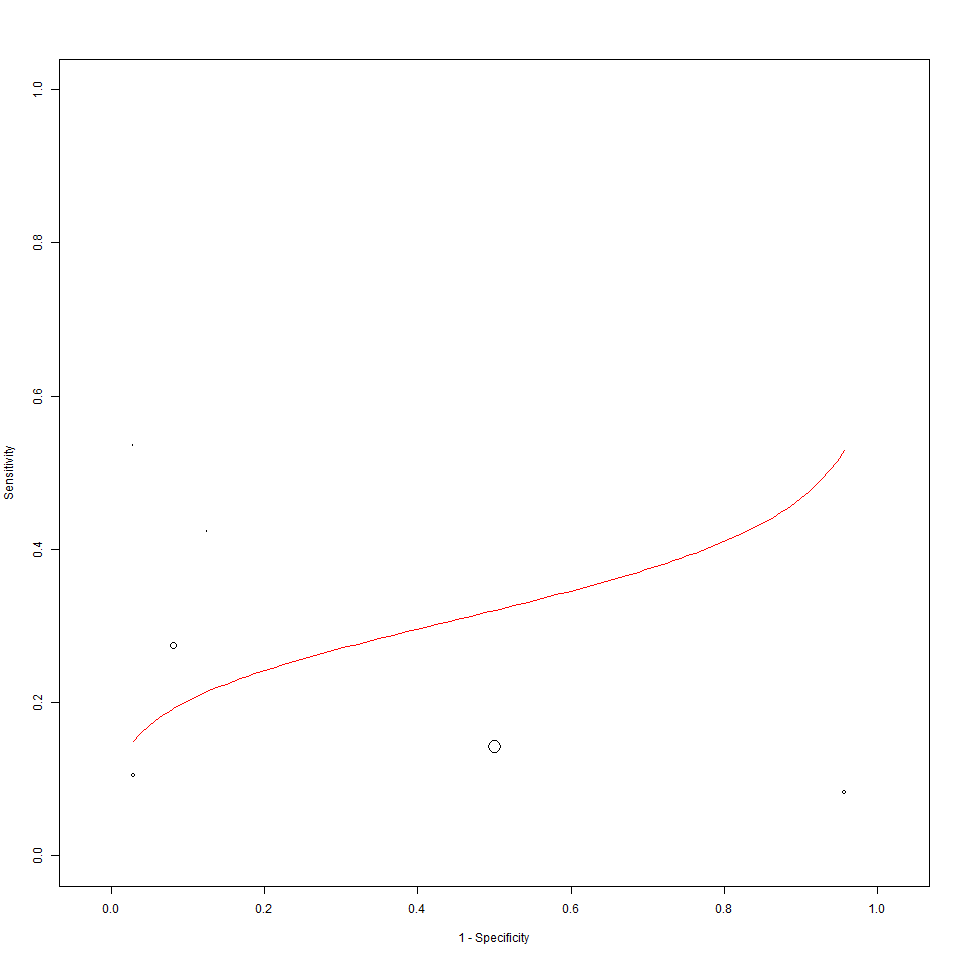


Figure S7 Placental exophytic mass A: ROC curve


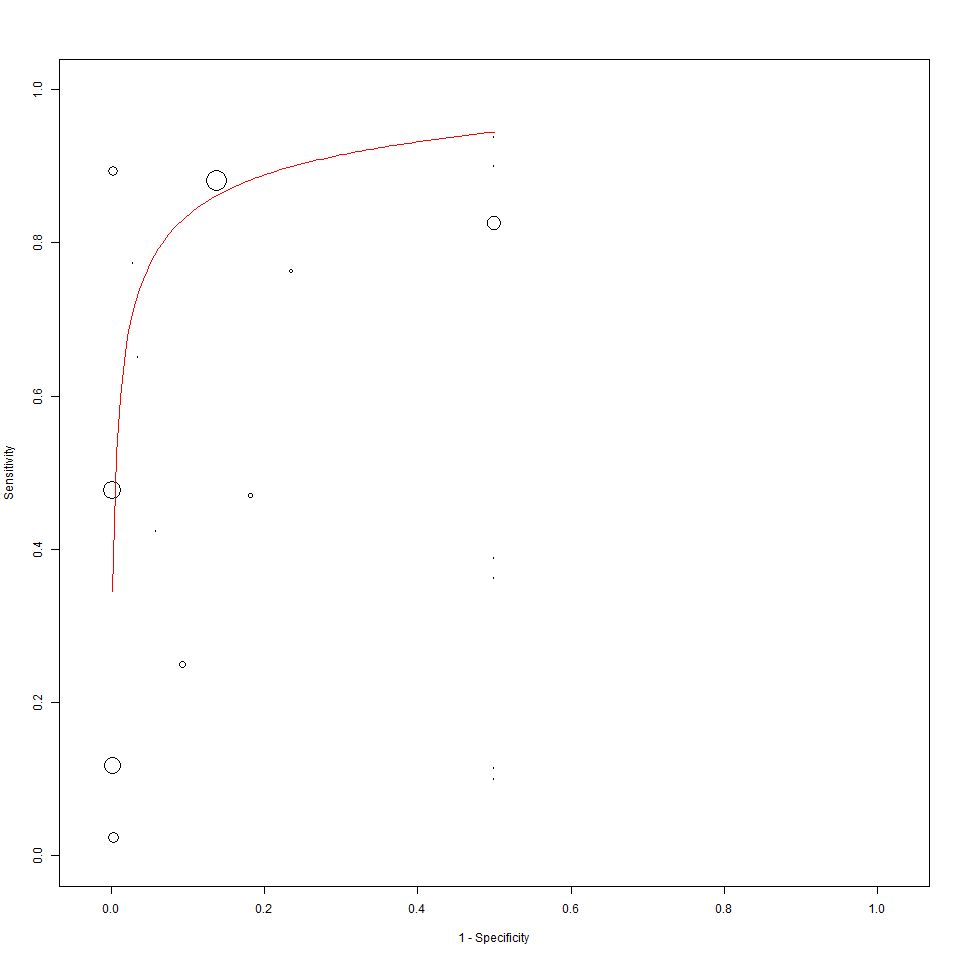


Figure S8 Uterovesical vascularity A: sensitivity and specificity, B Odd ratio, C NLR and PLR and D ROC curve


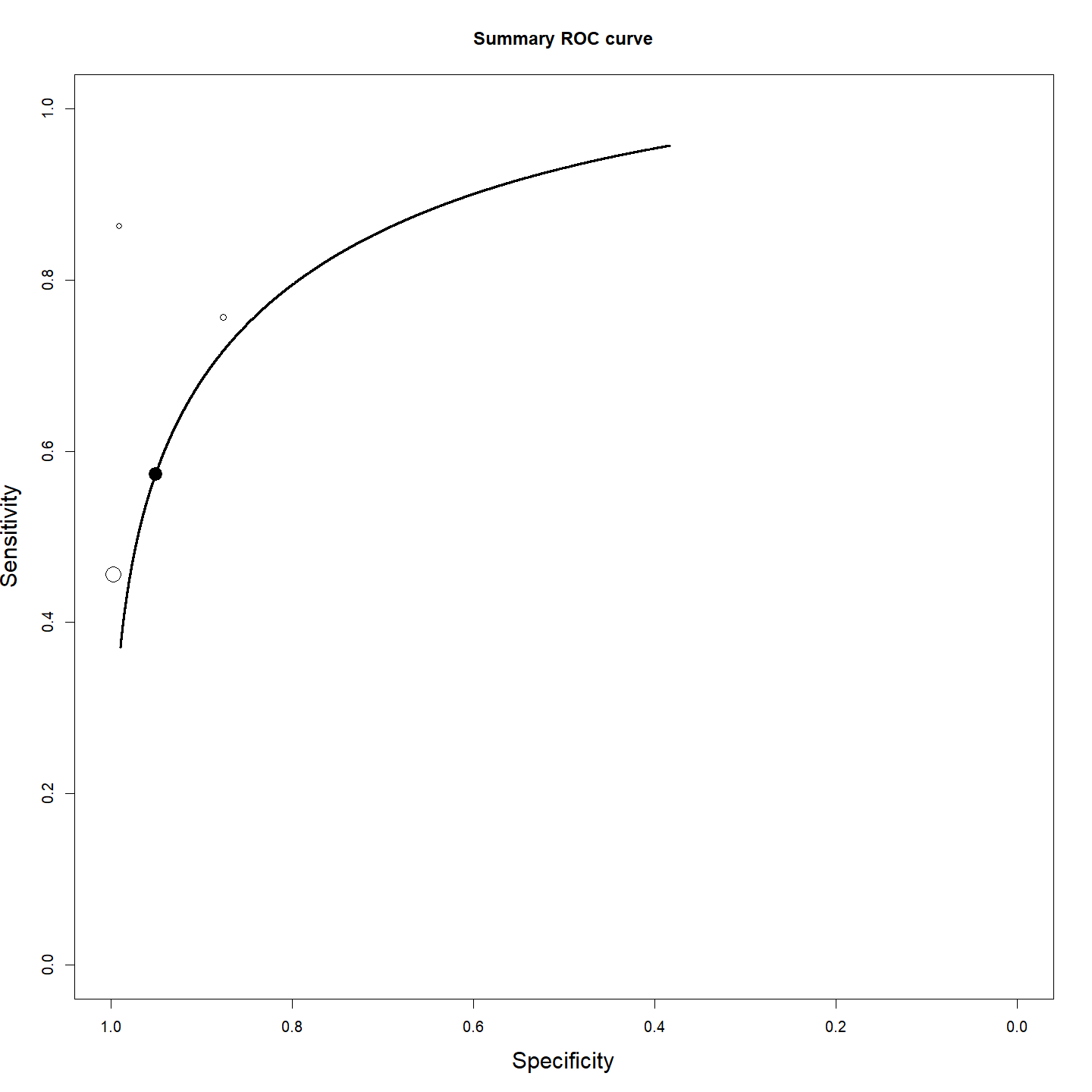


Figure S9 overall A: ROC of 3D ultrasound


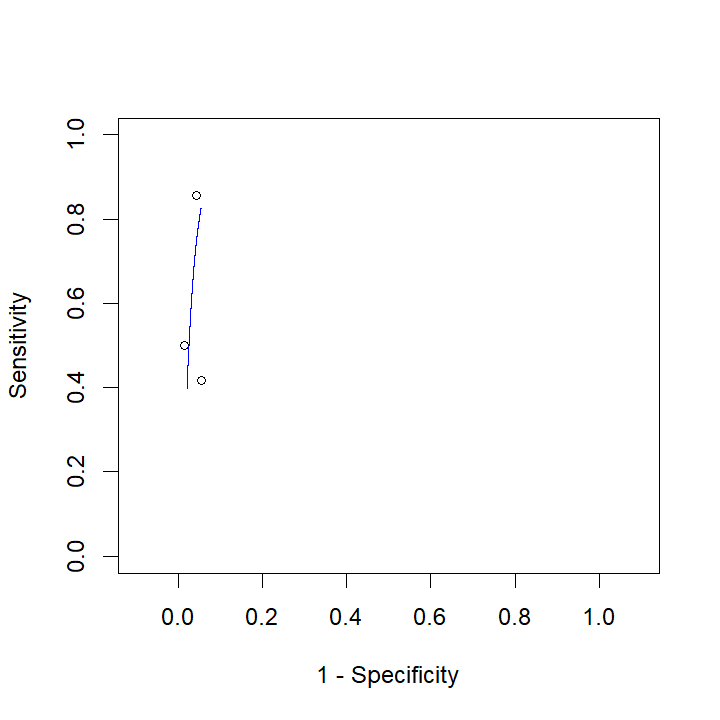


Figure S10 overall A: ROC of Posterior placenta
