# Supplementary material for: Diagnostic accuracy of ultrasound in the diagnosis of Placenta accreta spectrum: systematic review and meta-analysis
Source: BMC Pregnancy Childbirth. 2023 May 15;23:354. doi: 10.1186/s12884-023-05675-6 (PMC10186814; doi:10.1186/s12884-023-05675-6)
Supplement: Supplementary file 2 — Additional file 2. Supplementary tables [file 12884_2023_5675_MOESM2_ESM.docx]

Supplementary Tables

Table S1 General characteristics of the included studies

| Study name | settings | Type of study | Participants | diagnosis |
| --- | --- | --- | --- | --- |
| Abu Hashim 2021 | Single center Egypt | Prospective Cohort | 100 women  Inclusion criteria were: (1) pregnant women in the third-trimester;  (2) a single fetus (3) a previous delivery by  at least one CS; (4) an anterior PP  or anterior low-lying placenta.  Exclusion  criteria were women unwilling to participate or did not deliver at our hospital. | 23 PAS  8 accreta  7 Increta  8 percreta  77 Non adherent  Placenta accreta index score based on placental location,  number of CS, abnormal placental lacunae, sagittal smallest myometrial thickness, and bridging vessels. |
| AlAlfy 2021 | Single center Egypt. | Prospective Cohort | 90 pregnant women scheduled for an elective CD.  inclusion criteria: history of one or more previous CD or hysterotomy and have placenta  previa with the lower placental edge reaches the level of internal os or covering the cervix , gestational age 30–40 weeks. in the 3D-TUI , and 3D power Doppler ultrasound volume  was performed.  Exclusion:  Women with any medical disorders  BMI >40 kg/m2  cases presented to the emergency department due to unavailability of machines and fetal factors as multifetal pregnancy, IUGR, IUFD , premature rupture of membranes, and associated fetal anomalies | 54 PAS  16 accreta  24 increta  14 percreta  36 Non adherent  3D-TUI, and 3D power doppler |
| Alchalabi 2014 | Single center Jordan | Retrospective Case control | 81 women hospital who underwent (CS/hysterectomy) for PP/MAPP during the period March 2003–February 2012 was undertaken. Women  delivered by CD because of PP during the same period were control group. Women with low lying placenta were excluded. USS is routinely performed in  all women with PP and previous LSCS, MRI was performed in selected women. | 23 PAS  58 Non adherent |
| Algebally 2014 | Single center Qatar | Prospective Cohort | 100 women at a high risk of abnormal placentation with one or more of the following: placenta previa, previous uterine interventional procedures (e.g. CD, myomectomy or dilatation & curettage), maternal age of ≥ 35 years and grand multiparity | 100 participants  32 PAS  12 Accreta  8 Increta  12 percreta  68 Non adherent  Severity assessed  3D US, MRI |
| Ayati 2017 | Single center Iran | Cross sectional | 82 pregnant women with two or more of the following risk factors: placenta previa, prior uterine surgery (such as a previous CD, uterine curettage, or myomectomy), high maternal age (≥35 years), and high parity (≥5). Exclusion criteria were MRI contraindications, cardiac pacemaker, metal objects in the body, and patient’s refusal. | 82 participants  17 PAS  65 Non adherent  2D US , MRI |
| Balcacer 2016 | Single center  USA. | Retrospective study | 40 pregnant patients  underwent pelvic MRI for the evaluation of placental invasion based on clinical risk factors or findings on sonography.  Thirty-seven of the 40 patients had at least 1 prior US exam, the closest to the  date that MRI was performed was the one included for comparison  reference standards. | 18 PAS  22 Non adherent  Severity assessed  2D US, MRI |
| Bassetty 2021 | Single center India, | Cross sectional | Women with PAS were included.  Exclusion criteria women  with placenta previa only and if the placental biopsy was not available.  Women with co-existing medical disorders were not excluded . | 21 PAS  0 Non adherent  Diagnosis ,2D US,Doppler,MRI  Maternal morbidity |
| Borg 2018 | Single center Egypt. | Prospective cohort | 100 pregnant women with suspicious of PAS Inclusion criteria:  all pregnant women on third-trimester gestation with abnormal placental invasion including (patients with  placenta previa anteriorly located and attached on a  previous caesarian section scar, placenta attached on a previous uterine scar like myomectomy or hysterotomy  and history of placental retention during previous  delivery)  exclusion criteria include primigravida,  accidental hemorrhage, gestational age < 28  weeks, and if the placenta is located posteriorly. | 64 PAS  36 non adherent  2D ultrasonography, color  Doppler and MRI. |
| Bowman 2014 | Single center  USA. | Retrospective  Case-control | 111 women  Inclusion criteria for the study included: (1) clinical and/or pathologic diagnosis of placenta accreta or patients with  placenta previa but no accreta (matched by year of delivery); (2) delivery at the University of Utah Health Sciences Center  (3) sonography of the placenta available at a gestational age of 16 weeks or later.  Each sonographic study was independently reviewed, scored, and interpreted by 6 investigators.  the confidence of that diagnosis was scored for each imaging study on a scale of 0(none) to 10 (certain), and image quality was scored on a  scale of 1 (very poor) to 10 (best). | 55 PAS  56 Non adherent  6 sonographers  analyze interobserver variability of sonography |
| Budorick 2016 | Single center  USA | Retrospective case control | 45 patients with suspicion of placenta accreta based on clinical history, US findings or both. | 14 PAS  31 non adherent  2D US, Doppler, MRI |
| Cali 2013 | Italy | Prospective Cohort | 187 patients  placenta previa and history of uterine surgery and performed transabdominal and transvaginal ultrasound examination for early detection of PASr | 41 PAS  146 Non adherent  2D US,3D US ,Doppler |
| Chong 2018 | Single center  China | Prospective cohort | 180 patients with  suspected PAS (mostly patients with previous cesarean and placenta previa and  the other patients were placenta previa with >2 times abortions);  assessed by ultrasound scoring system before delivery. Exclusion criteria: patients  diagnosed as abnormal during early pregnancy were terminated;  patients who have high risk complications, but her clinical data  were incomplete; and pregnancy not terminated in hospital. | 115 Accreta  38 increta  27 percreta  2D US, Doppler  Scoring |
| Chou 2000 | Single center  China | Prospective cohort | 80 patients with persistent placenta previa in the  second and third trimesters | 14 PAS  66 Non adherent  2D US, Doppler |
| Comstock 2004 | Single center  USA | Prospective Cohort | All patients with a previous cesarean delivery and an anterior placenta or placenta previa were evaluated | 15 PAS  8 accreta  3 Increta  4 percreta  18 Non adherent  2D US, Doppler |
| Davutoglu 2018 | Single center  Turkey | Prospective cohort | 29 patients with anteriorly localized placenta previa and having at least one risk factor for placenta accreta, as previous CD, curettage or myomectomy. Exclusion criteria included taking medication, smoking, high blood pressure, preeclampsia, gestational diabetes mellitus, multiple gestations, contraindication for MRI (presence of metallic implants, claustrophobia), and fetal chromosomal or structural anomalies | 13 PAS  16 Non adherent  2D US, MRI |
| de Marcillac 2016  Article in French | Single center  France | Retrospective | Twenty-two potential cases of placenta accreta (patients with an anterior placenta praevia with history of scarred uterus) | 13 PAS  9 Non adherent  2D US, MRI |
| Dwyer 2008 | 3 centers  USA | Retrospective | patients who underwent  both prenatal transabdominal sonography and MRI to evaluate for suspected diagnosis of  PAS. | 15 PAS  17 non adherent 2D US, Doppler, MRI |
| El Wakeel 2017 | Single center  Egypt | Both Prospective and Retrospective | 20 cases  Inclusion criteria  • Pregnant women with persistent placenta  previa (after 28 weeks of gestation), with  implantation on the lower uterine segment  • Previous uterine surgery (e.g., CD,  myomectomy, or fractional curettage).  Exclusion criteria  • Medical disorders (diabetes,  hypertension, epilepsy)  • Presence of metallic prosthesis. | 11 PAS  9 Non adherent  2D US, Doppler, MRI |
| Elhawary 2013 | Single center  Egypt | Prospective cohort | 39 pregnant women-  with placenta previa.  Such cases were at high risk for placenta accreta regarding their clinical history of either  one or all of the following: (1) maternal age > 35 years, (2) grand multiparity, (3) previous  uterine interventional procedures (e.g. CD, dilatation & curettage and  myomectomy) in addition to the major co-existing factor which is placenta previa. | 8 PAS  31 Non adherent  2D US, Doppler, MRI |
| Finberg 1992 | USA | Prospective cohort | 34 patients  had one or more CD and who were found to have placenta  previa with a portion of the placenta extending along  the anterior lower uterine wall. | 15 PAS  19 Non adherent  2D US, Doppler |
| Fitzpatrick 2013 | 221 centers  UK | Prospective Cohort | 133 women identified as having placenta  accreta, increta or percreta  diagnosed histologically following  hysterectomy, or post-mortem, or an abnormally adherent  placenta, requiring active management, including conservative approaches where the placenta is left in situ. | 133 PAS  0 Non adherent  2D US, Doppler, MRI |
| Garofalo 2019 | Single center  Italy | Prospective cohort | 198 women.  Inclusion criteria: more than 26 weeks’ gestation; placenta localized mainly (more than 50% of its whole  part) on the uterine posterior wall; placenta was defined as previa  when it covered the internal os and low-lying when the placental  edge was <20 mm from the internal os | 20 PAS  11 accreta  7 increta  2 percreta  178 non adherent  2D US, Doppler  Posterior placenta only |
| Guo 2021 | Single center China | Prospective Cohort | 70 women with singleton pregnancy during 2^nd^ or 3^rd^ trimester of pregnancy; no metal implants in their body; physical condition met the criteria for MRI and ultrasound; conscious with normal communication ability. Exclusion criteria: state of tension, anxiety or tachypnea; obvious fetal movements, which affected the diagnosis effects; other diseases that seriously affected the results; refusal to cooperate with the study | 45 PAS  25 Non adherent |
| Hamada 2011 | Single center  Japan | Retrospective case control | A case-control study with 1: 5 matched pairs. cases with placenta previa at the time of delivery were retrieved from medical records. Control  cases with normal placental location at the time delivery were also included in the study. Each pair was matched for maternal age  Subjects with multiple pregnancies and stillbirths were excluded | 5 PAS  3 Accreta  2 increta  65 Non adherent  2D US, Doppler |
| Hamisa 2015 | Single center  Egypt | Prospective cohort | 120 women that have or gave history of third trimester bleeding. Those had repeated cesarean sections. | 4 PAS  116 non adherent  2D US, Doppler, MRI |
| Japaraj 2007 | Three centers  Malaysia | Prospective cohort | 21 women with  previous caesarean scar, partial or total placenta previa diagnosed after 28 weeks gestation  and they had to be hemodynamically stable at the time of the ultra-  sound examination to determine the abnormal placentation. Patients who were actively bleeding were  excluded. | 7 PAS  14 Non adherent  2D US, Doppler |
| Knight 2018 | Single center  USA | Retrospective Cohort | 41 pregnant women who received transabdominal and transvaginal US and MRI for concerns of morbidly adherent placenta | 16 PAS  25 Non adherent  Score |
| Kumar 2016 | Single center  India | Prospective cohort | 22 patients referred for sonography for evaluation of invasive placenta, mainly due to a previous history of CD or other uterine surgeries. | 9 PAS  13 non adherent |
| Lerner 1995 | Single center  USA | Prospective cohort | 21 patients with persistent placenta previa in third trimester | 5 PAS  16 non adherent  2D US, Doppler |
| Lim 2011 | Single center  USA | Retrospective case control | 13 patients with risk factors for placenta accreta. Only patients who underwent both  pelvic ultrasound and MRI were considered in this study. | 9 PAS  5 accreta  3 increta  1 percreta  4 non adherent  2D US, Doppler, MRI |
| Lopes 2019  Article in Portuguese | Single center  Brazil | Retrospective  Cohort | 37 women with diagnoses of placenta previa who had been examined due to complaints of transvaginal bleeding | 16 PAS  9 accreta  6 increta  1 percreta  21 Non adherent  Separate analysis for ant and post located placenta |
| Luo 2019 | Single center  China | Prospective cohort | 392 Patients aged 19–44 years who had sonographic confrmation of placenta previa after 28 weeks of gestation were the subjects of  this study. We excluded those women who experienced an  emergency referral and subsequent surgery due to massive vaginal bleeding without routine ultrasonographic examination in our hospital. | 160 PAS  79 accreta  53 increta  28 Percreta  232 Non adherent  2D US, Doppler  score |
| Maged 2018 | Single center  Egypt | Prospective | 100 patients with singleton pregnancy, PP anterior (marginalis anterior, incomplete centralis, or complete centralis) with at least one previous CS, and the gestational age > 28 weeks. Exclusion criteria were patients with unscarred uterus, or scared uterus rather than CS, patients with normally situated placenta, and PP  posterior those with coagulopathy. | 63 PAS  32 Accreta  22 Increta  9 Percreta  37 non adherent  2D US, Doppler |
| Magied 2018 | Single center  Egypt | Prospective Cohort | 23 pregnant females presenting with placenta previa and risk factors as; mother’s age exceeding 35 years, having many offspring and prior interventional procedures of the uterus for instance CD, dilatation & curettage | 11 PAS  6 accreta  2 increta  3 percreta  12 non adherent |
| Maher 2013 | Single center  KSA | Prospective cohort | 577 Patients with placenta previa>28 weeks  regardless of age or parity.  If full delivery data were not available, the patient was excluded, as were patients who delivered outside the unit. | 42 PAS  553 non adherent  2DUS, Doppler, MRI |
| Mansour 2011 | Single center  Egypt | Prospective cohort | 36 cases at high risk for placenta accreta regarding their clinical history of either one or all of the following: (1) maternal age > 35 -  years, (2) grand multiparity, (3) previous uterine interventional  procedures (e.g. CD, dilatation & curettage and myomectomy) in addition to the major co-existing factor  which is placenta previa. | 15 PAS  21 non adherent  2DUS, Doppler, MRI |
| Marsoosi 2018 | 3 centers Iran | Prospective cohort | 159 patients with previous cesarean delivery who fulfilled 1 or more of the  following criteria: (1) History of curettage, myomectomy or other uterine surgery and (2) Gestational age greater than or equal 18 weeks. | 49 PAS  110 non adherent  2D US, MRI  Score |
| Masselli 2008 | Italy | Prospective cohort | 50 Patients in third trimester of pregnancy with diagnosis of placenta previa and at least one previous caesarean section. women for whom complete  information was available regarding clinical and pathological diagnosis were included in this study. | 12 PAS  38 non adherent  2D US, Doppler, MRI |
| Morel 2021 | Multicenter in 15 countries (14 European and USA) | Both prospective and retrospective | 318 patients with PAS from all participating centers were included and none excluded and only expert imagers in the field of PAS included  cases. The patients reported were not chosen. All the cases included in the database for which antenatal diagnosis has been achieved were analyzed in this study. The 95 cases for which data was missing or that had been discovered at the time of delivery were excluded. | 318 PAS  105 Accreta/Increta  213 Percreta  MRI done in 119 PAS  (36 Accreta/Increta  83 Percreta)  0 non adherent  2D US, Doppler, MRI |
| Nawab 2017 | Sigle center  Pakistan | Prospective cohort | 25 antenatal patients who were at high clinical risk of placenta accreta were identified and undergone USG and MRI | 7 PAS  18 Non adherent |
| Peker 2013 | Single center Turkey | Prospective case control | 40 patients with the diagnosis of total placenta previa who had reached 28 weeks of pregnancy or more | 20 PAS  10 accreta  7 increta  3 percreta  20 Non adherent |
| Pilloni 2016 | Single center  Italy | Prospective cohort | 314 pregnant women with persistent placenta previa (after  26 weeks’ gestation), who delivered subsequently at Sant’  Anna Hospital | 37 PAS  277 non adherent  2D US, Doppler  Separate analysis for anterior and posterior placenta |
| Rac 2015 | Single center  USA | Retrospective | 184 pregnantas with 1 prior cesarean delivery who had sonographic confirmation of  placenta previa or low-lying placenta in  the third trimester and were delivered at our hospital. | 54 PAS  130 Non adherent  Placenta accreta index |
| Rekawek 2022 | Single center  USA | Retrospective cohort | 41 patients with evidence of PAS on prenatal ultrasound | 15 PAS  26 Non adherent |
| Rezk 2014 | Single center  Egypt | Prospective cohort | 74 women with  persistent placenta previa at 32–34 weeks, with previous uterine scar (CD and/or myomectomy),  hemodynamically stable,  Patients who were  actively bleeding or had ultrasonographic diagnosis of uteri  with congenital anomalies were excluded. | 53 PAS  21 non adherent |
| Riteau 2014 | 2 centers France | Retrospective | 42 patients  referred for suspected placenta accreta, study population included 42 pregnant women who had been investigated by both ultrasound  and prenatal MRI. | 26 PAS  16 non adherent |
| Romero 2021 | Single center  Italy | Retrospective cohort | 70 pregnant patients with PP who underwent US and MRI examinations at our institution for suspicion of PAS | 24 PAS  14 Accreta  7 Increta  3 Percreta  46 Non adherent |
| Shih 2009 | Taiwan/ Argentina | Prospective cohort | 170  pregnant women with persistent placenta previa (after  28 weeks’ gestation) were prospectively enrolled into this  study. The inclusion criteria were: complete imaging using all diagnostic techniques (gray-scale, color Doppler and 3D power Doppler), and full availability of delivery information. | 39 PAS  131 non adherent  2D US, 3D US, Doppler |
| Shweel 2012 | Single center  Egypt | Prospective cohort | 28 pregnant women with a history of previous uterine surgery and  suspected to have PAS. Inclusion criteria: (1) Abnormal placental position (total, marginal,  low-lying. (2) One of the following transabdominal sonographic gray scale findings  that suspects placenta accreta (presence of placental lacunae,  loss of the retro-placental sonolucent zone, and thinning or  disruption of hyperechoic serosa–bladder interface). | 11 PAS  17 non adherent  2D US, Doppler, MRI |
| Twickler 2000 | USA | Prospective | 20 women with a history  of previous Cesarean delivery who had third trimester  bleeding, or who were scheduled for repeat Cesarean, whose placentas were anterior, or previa, including anterior low  lying based on  transvesical pelvic real-time imaging. | 11 PAS  3 accreta  2 increta  4 percreta  9 non adherent  2D US, Doppler |
| Warshak 2006 | Single center  USA | Retrospective | 40 women with placenta previa,  low-lying placenta with a previous cesarean delivery,  or myomectomy evaluated by ultrasonography.  suspicious or inconclusive findings were evaluated by MRI.  Inclusion was limited to those for whom complete clinical and pathologic information. | 26 PAS  14 Non adherent  2D US, Doppler, MRI |
| Xia 2021 | China | Retrospective | 245 pregnant women with suspected placenta accreta were evaluated by abdominal  ultrasound and MRI, 86 placenta accreta patients were  ultimately determined by clinical and pathologic diagnosis. | 86 PAS  159 non adherent  2D US, Doppler, MRI |
| Zhou 2014 | China | Prospective | 68 singleton pregnant women with a history of one or more CDs Exclusion criteria were preeclampsia, intrauterine  growth restriction, fetal aneuploidy, use of tocolytic agents, and uterine bleeding at or after blood sampling because these complications could alter the level of cell-free  placental mRNA | 33 placenta previa  12 PAS  21 Non adherent  2D US, Doppler, MRI, cell-free β-HCG mRNA |

Table S2 GRADE quality of evidence

| Outcome | No studies | Risk of bias | Inconsistency | Indirectness | Imprecision | | Publication bias | Quality |
| --- | --- | --- | --- | --- | --- | --- | --- | --- |
|  |  |  |  |  | Sample size | Wide CI |  |  |
| Overall estimate | 50 | N | S | N | 4841 | N | N | Moderate |
| Myometrial thinning | 17 | N | S | N | 1749 | N | N | Moderate |
| Loss of retroplacental clear zone | 25 | N | S | N | 2016 | N | N | Moderate |
| Bridging vessels | 12 | N | S | N | 873 | N | N | Moderate |
| Placental lacunae | 35 | N | S | N | 2868 | N | N | Moderate |
| Bladder wall interruption | 19 | N | S | N | 1900 | S | N | Low |
| Exophytic mass | 6 | N | S | N | 586 | S | N | Low |
| Uterovesical vascularity | 18 | N | S | N | 2193 | N | N | Moderatea |

CI Confidence Interval; N Not serious; S Serious
